# Pharmacological activation of p53 induces dose-dependent changes in endothelial cell fate during angiogenic sprouting

**DOI:** 10.1038/s41419-025-08292-7

**Published:** 2025-12-08

**Authors:** Omayma Al-Radi, Katrine Ingelshed, Lisa Eichhorn, Heidi Josefsson, Martin Krkoska, Lars Bräutigam, Susanne Lindström, Ákos Végvári, Sania Kheder, Carmine P. Cerrato, Suzon Fermé, Cecilia Bosdotter, Amin Allalou, Fredrik Levander, Borivoj Vojtesek, David P. Lane, Pavitra Kannan

**Affiliations:** 1 Department of Microbiology, Tumor and Cell Biology, Karolinska Institutet, Stockholm, Sweden; 2 Department of Immunology, Genetics and Pathology, Uppsala University, Uppsala, Sweden; 3 RECAMO, Masaryk Memorial Cancer Institute, Brno, Czech Republic; 4 Comparative Medicine, Karolinska Institutet, Stockholm, Sweden; 5 Department of Medical Biochemistry and Biophysics, Karolinska Institutet, Stockholm, Sweden; 6 Department of Information Technology, Uppsala University, Uppsala, Sweden; 7 DanioReadout, Immunology Genetics and Pathology, Uppsala University, Uppsala, Sweden; 8 SciLifeLab BioImage Informatics Facility, Uppsala University, Uppsala, Sweden; 9 Department of Immunotechnology, National Bioinformatics Infrastructure Sweden, Science for Life Laboratory, Lund University, Lund, Sweden

**Keywords:** Angiogenesis, Pharmacology, Proteomics, Tumour-suppressor proteins, Cell biology

## Abstract

The cell cycle is a key regulator of endothelial cell specification into tip and stalk cell phenotypes, which are essential for angiogenesis in both normal development and pathological conditions. While the tumor suppressor p53 is known to regulate the cell cycle and influence cell fate, its role in modulating the cell fate of these phenotypes remains unclear. Using non-genotoxic small molecule and stapled peptide compounds to pharmacologically activate p53 via MDM2 inhibition, we demonstrate that graded levels of p53 induce distinct cellular fates in normal endothelial cells. Low levels of p53 induce reversible cell cycle arrest by reducing DNA replication, while high levels induce senescence and cell death. Surprisingly, all tested levels of p53 activation reduced the growth of venous blood vessels in vitro and in zebrafish embryo models. This reduction in sprouting may stem from distinct cellular responses in tip-like and non-tip-like cells to pharmacological p53 activation: low p53 levels primarily reduced proliferation in non-tip-like cells, whereas high levels decreased the frequency of tip-like cells and the expression of genes associated with tip and stalk cell identities. Our findings show for the first time that pharmacological p53 activation modulates endothelial cell fate in a dose-dependent manner during sprouting angiogenesis. They also highlight the potential of using graded p53 modulation as a therapeutic strategy to target abnormal tip or stalk cell development in pathological angiogenesis, such as in cancer.

## Introduction

Angiogenesis is a fundamental biological process during which endothelial cells undergo several phenotypic transitions to form new blood vessels from existing vessels [1, 2]. When exposed to growth stimuli, a single tip cell extends from the existing vessel and migrates toward the growth signal using filopodia, while adjacent stalk cells elongate the sprout [3]. The specification of tip and stalk cell phenotypes is carefully regulated during development and tissue repair. However, it becomes dysregulated in cancer, age-related macular degeneration, and ischemic conditions, leading to excessive or insufficient vessel growth that can enable tumor growth or cause tissue damage, respectively. Understanding the mechanisms that regulate the specification of endothelial cells into these phenotypes is crucial for improving treatment strategies for these diseases [3].

Preclinical studies have shown that the cell cycle plays a critical role in tip and stalk cell specification during angiogenesis [4, 5]. In zebrafish embryo models of sprouting angiogenesis, tip cells emerge from the dorsal aorta in S/G2/M phases [6] or from the posterior cardinal vein in G1 phase [7]. Vessel growth subsequently occurs through tip cell division, stalk cell proliferation, or tip and stalk cell proliferation [6–9]. In contrast, these mechanisms differ in mouse retinal models of sprouting angiogenesis. Tip cells emerge from the G1 phase and rarely divide, while stalk cells proliferate at the angiogenic front [10]. Despite the differences between these models, chemical or genetic disruption of cell cycle phases leads to vascular abnormalities in both models [6–10], indicating that cell cycle regulators are integral to specification of endothelial cell phenotypes during angiogenesis.

A key regulator of cell cycle progression is the tumor suppressor protein p53. It is kept at low levels through proteosomal degradation under unstressed conditions. However, p53 is stabilized under stressed conditions, resulting in the transcriptional activation of its downstream targets that mediate cell cycle arrest, senescence, or apoptosis [11]. While p53 deletion in endothelial cells does not impair vascular development [12], its activation in normal endothelial cells has been shown both to promote the initiation of sprouting through increased cell cycle arrest [7] and to reduce sprouting through decreased proliferation [13] or enhanced apoptosis [14]. However, in vascular diseases, the activation of p53 in endothelial cells appears to be primarily associated with decreased vessel formation [15]. In cancer and other normal cells, the level of p53 activation can lead to distinct cell fates [16]. In the context of angiogenesis, these data raise the possibility that p53 levels may determine angiogenic outcome, and/or may differentially affect tip and stalk cells. Yet, the effects of p53 activation on the fate of these endothelial phenotypes during sprouting angiogenesis are not known.

We hypothesized that different levels of p53 influence endothelial cell fate by triggering distinct responses in tip vs stalk cells, thereby resulting in varied phenotypic effects during angiogenesis. Specifically, we predicted that low levels of p53 activation could promote the initiation of sprouting by inducing cell cycle arrest, thereby increasing the proportion of tip cells. In contrast, we predicted that high levels of p53 activation could reduce sprouting by inducing cell death in both tip and stalk cells. To test this hypothesis, we studied the molecular and phenotypic effects by which different levels of p53 activation affect endothelial cells using cellular assays, proteomics, and in vitro and in vivo sprouting assays. We used a pharmacological approach to induce different levels of p53 by applying graded concentrations of a new generation of small molecule and stapled peptide compounds that transcriptionally activate p53 via inhibition of its negative regulator MDM2 [17–21]. Our findings reveal that different levels of p53 activation trigger distinct cellular responses in each population, but surprisingly lead to the same angiogenic outcome of reducing vessel growth.

## Results

### Graded p53 activation by MDM2 inhibitors induces distinct cell fates in endothelial cells

**Fig. 1:**
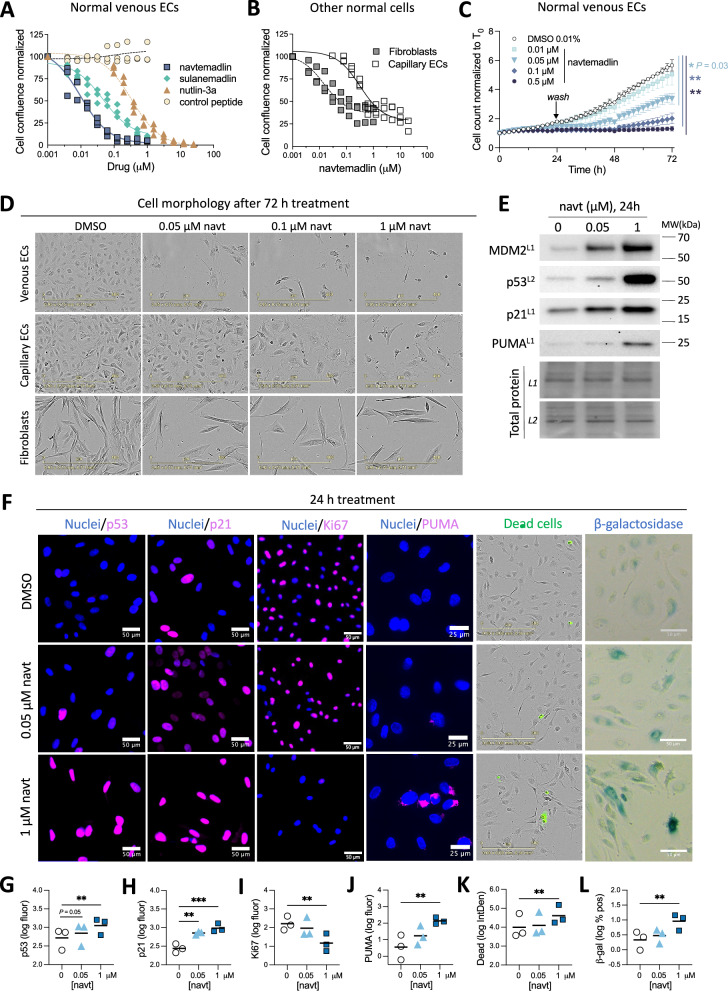
Pharmacological activation of p53 induces cell cycle arrest, cell death, and senescence in endothelial cells in a concentration-dependent manner. **A** Concentration-dependent growth inhibition of human umbilical vein endothelial cells (HUVEC) by three p53-activators (MDM2 inhibitors navtemadlin and nutlin-3a; MDM2/MDMX inhibitor sulanemadlin), but not by non-specific control peptide. Cell growth was measured by live-cell imaging as the percent confluence normalized to untreated wells following 72 h treatment. Data points show averaged value from one experiment (*n* = 3 experiments) and are fitted with a best fit model for concentration-growth response. **B** Growth inhibition of human dermal microvascular endothelial cells (HDMEC) and normal human dermal fibroblasts (NHDF) by navtemadlin, as measured by live-cell imaging. Data points show averaged value from one experiment (*n* = 3 experiments) and are fitted with a best fit model for concentration-growth response. **C** Growth inhibition of HUVEC recovers within 48 h following an initial 24 h treatment using ≤ 0.1 μM navtemadlin. Cell growth was measured by live-cell imaging and quantified over 72 h as cell counts per well and normalized to counts at time 0. Data points show mean  ± SD (*n* = 3 independent experiments). *P_*adj*_ < 0.05, **P_*adj*_ < 0.01 using 1-way, repeated measures ANOVA with adjustment using Dunnett’s correction. **D** Morphological abnormalities are visible in phase-contrast images of venous ECs (HUVEC) and capillary ECs (HDMEC), but not in those of fibroblasts (NHDF) after 72 h of navtemadlin treatment, but not after 24 h. Scale bar = 200 μm. **E** Increased expression of proteins involved in p53 signaling (MDM2, clone IF2, 0.5 μg/mL; p53, clone DO-1, 0.4 μg/mL), cell cycle arrest (p21, clone 12D1, 0.24 μg/mL), and apoptosis (PUMA, clone D30C10, 0.96 μg/mL) in HUVEC following 24 h navtemadlin treatment, as determined by western blot analysis. Total protein controls correspond to distinct membranes (L1 or L2). Images are cropped from full-length blots of one biological experiment (see ‘Full Length Western Blots’) and are representative of at least two experiments. Further details regarding antibodies used are shown in SI Table 1. **F** Increased expression of p53 (clone DO-1, 12 μg/mL), cell cycle arrest (p21, clone 12D1, 1.22 μg/mL), apoptosis (PUMA, clone D30C10, 4.8 μg/mL), dead cells (Sytox green, 100 nM), and senescence (β-galactosidase), and reduced expression of active cell cycle (Ki67, clone SP6, 0.12 μg/mL), following 24 h treatment of HUVEC using navtemadlin as visualized by immunofluorescence, live-cell fluorescence imaging, and colorimetric staining. Scale bar = 50 μm. Further details regarding antibodies and concentrations used are shown in SI Table 1. **G–K** Quantification of fluorescence and colorimetric levels of protein markers, showing increased expression of p53, cell cycle arrest (p21), apoptosis (PUMA), cell death (Sytox green), and senescence, and reduced activity in cell cycle (Ki67). Data points indicate value from one experiment (*n* = 3 experiments). **P_*adj*_ < 0.01, ***P_*adj*_ < 0.001 using one-way ANOVA with adjustment using Dunnett’s correction. Horizontal black line indicates the mean value.

The molecular consequences of activating p53 in normal endothelial cells were investigated using two small molecule MDM2 inhibitors and one stapled peptide MDM2/MDMX inhibitor, which stabilize p53 by preventing it from being targeted for degradation by MDM2. Cellular growth of human umbilical vein endothelial cells (HUVEC) decreased by nearly 100% in a concentration-dependent manner upon treatment with the small molecules navtemadlin and nutlin-3a, and the stapled peptide sulanemadlin (Fig. 1A). Cellular growth was more potently inhibited by navtemadlin (IC_50_ = 0.010 µM [95% CI = 0.007–0.013]) than by nutlin-3a (IC_50_ = 0.340 µM [95% CI = 0.266–0.413]) or sulanemadlin (IC_50_ = 0.046 µM [95% CI = 0.028–0.064]). In contrast, treatment with a control stapled peptide did not affect HUVEC growth at any concentration (Fig. 1A). The growth-reducing effects were not specific to HUVEC, as growth reductions of 60–75% after navtemadlin treatment were also observed in normal human dermal microvascular endothelial cells (IC_50_ = 0.319 µM [95% CI = 0.171–0.468]) and normal human dermal fibroblasts (IC_50_ = 0.015 µM [95% CI = 0.006–0.024], Fig. 1B). Since navtemadlin was the most potent inhibitor among those tested, it was used in subsequent experiments.

Within the experimental time frame, the growth-reducing effects induced by p53 activation in HUVEC could be recovered at the population level at low concentrations, but not at high concentrations of navtemadlin treatment (Fig. 1C). While HUVEC treated with 0.01 µM navtemadlin for 24 h had no significant effects on regrowth (*P*_*adj* at 72h_ = 0.70), cells treated with 0.05 µM (*P*_*adj* at 72h_ = 0.03) or 0.1 µM navtemadlin (*P*_*adj* at 72h_ = 0.007) for 24 h had significant delays in regrowth. In contrast, cells treated with 0.5 µM navtemadlin for 24 h did not regrow in the experimental time frame (Fig. 1C). Moreover, in brightfield images of HUVECs, increasing concentrations of navtemadlin were associated with cell rounding after 72 h (Fig. 1D), indicative of cell death. Cellular rounding was also observed after 72 h treatment in dermal microvascular endothelial cells, but not in dermal fibroblasts (Fig. 1D), highlighting the remarkable sensitivity of endothelial cells to pharmacological p53 activation.

We reasoned that the observed morphological changes in endothelial cells after navtemadlin treatment might reflect a switch from cell cycle arrest to cell death. To capture the molecular changes that precede morphological changes, we measured the expression of key proteins in the p53 pathway following 24 h treatment with low (0.05 μM) and high (1 μM) concentrations of navtemadlin. In western analysis of HUVEC lysates, the expression of MDM2 (negative regulator of p53), p53, and p21 (marker of cell cycle arrest) proteins each increased more than 1.7-fold at 0.05 μM and more than 4-fold at 1 μM. In contrast, the expression of p53-upregulated modulator of apoptosis (PUMA) did not measurably increase at 0.05 μM but increased by 4-fold at 1 μM (Fig. 1E). Similar results were found using immunofluorescence staining in HUVECs. The expression of p53 increased at 0.05 μM with weak evidence (*P*_*adj*_ = 0.05) and at 1 μM with strong evidence (*P*_*adj*_ = 0.002), while p21 increased at both concentrations (Fig. 1F–H; *P*_*adj* for 0.05 μM_ = 0.002; *P*_*adj* for 1 μM_ = 0.0006). The expression of Ki67 (marker of proliferation found in active phases of the cell cycle) decreased only at 1 μM (Fig. 1F, I; *P*_*adj* for 0.05 μM_ = 0.26; *P*_*adj* for 1 μM_ = 0.003); given that Ki67 levels decline gradually during G_0_/G_1_ arrest as a function of time, transient decreases in proliferation may not be reflected by a measurable drop in Ki67 levels at 0.05 μM [22, 23]. Treatment with 1 μM navtemadlin was also associated with increased cell death as measured by increased PUMA expression (Fig. 1F, J; *P*_*adj* for 0.05 μM_ = 0.11; *P*_*adj* for 1 μM_ = 0.008) and by live imaging of dead cells (Fig. 1F, K; *P*_*adj* for 0.05 μM_ = 0.43; *P*_*adj* for 1 μM_ = 0.003), as well as with induction of senescence as measured by β-galactosidase (Fig. 1F, L; *P*_*adj* for 0.05 μM_ = 0.18; *P*_*adj* for 1 μM_ = 0.001). Taken together, these results suggest that the low concentrations of pharmacological p53 activation in endothelial cells mainly result in cell cycle arrest, while higher concentrations result in cell death and senescence.

### The cellular effects induced by navtemadlin are largely p53-dependent

**Fig. 2:**
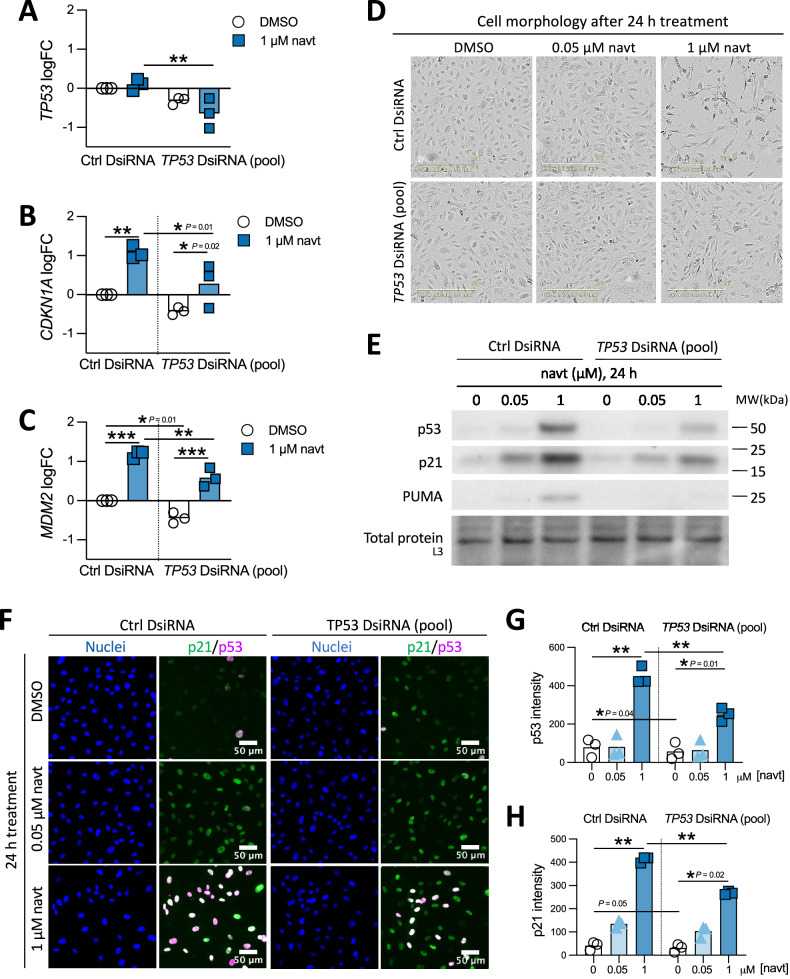
Knockdown of *TP53* reduces activation of p53 pathway induced by navtemadlin in HUVEC. **A–C** Knockdown of *TP53* reduces the activation of *TP53* and its target genes *CDKN1A* and *MDM2* induced by 6 h treatment with 1 μM navtemadlin in HUVEC, as measured by RT-qPCR. Cells were transfected for 24 h using Dicer-substrate short interfering RNA (DsiRNA) targeting a negative control sequence (“Ctrl”) or a pool of three DsiRNAs targeting *TP53* before navtemadlin treatment in fresh medium. Data points represent values from one experiment (*n* = 3 independent experiments) and are plotted as log_10_ fold change (FC). Bar height indicates mean value. Statistical analysis was performed on the delta Ct values, after normalization to housekeeping gene *B2M*. *P_*adj*_ < 0.05, **P_*adj*_ < 0.01, ***P_*adj*_ < 0.001 using two-way ANOVA with adjustment using Šidák’s correction. **D** Phase-contrast images of HUVEC following knockdown of *TP53* show phenotypic rescue of morphological changes observed after 24 h treatment using 1 μM navtemadlin. Scale bar = 300 μm. **E** In HUVEC treated with *TP53* DsiRNA, protein levels of p53, p21, and PUMA are reduced following 24 h navtemadlin, as determined by western blot analysis. Images are cropped from full-length blots of one biological experiment (see ‘Full Length Western Blots’) and are representative of at least three experiments. **F** In HUVEC treated with *TP53* DsiRNA, protein levels of p53 and p21 are reduced following 24 h navtemadlin treatment, as visualized by immunofluorescence staining. Scale bar = 50 μm. **G**, **H** Quantification of fluorescence levels of p53 (**G**) and p21 (**H**) shows reduced expression in HUVEC treated with *TP53* DsiRNA compared to those treated with control DsiRNA. Data points indicate averaged value from one experiment (*n* = 3 experiments). *P_*adj*_ < 0.05, **P_*adj*_ < 0.01, ***P_*adj*_ < 0.001 using two-way ANOVA with adjustment using Dunnett’s correction. Bar height indicates mean value. Statistical analysis was performed on log-transformed data, but plotted on linear scale to show differences more clearly.

To test whether the molecular and phenotypic changes induced by navtemadlin are p53-dependent, we performed molecular assays using HUVECs transfected with Dicer-substrate siRNA (DsiRNA) targeting *TP53* or a non-human control sequence. Since the phenotypic changes observed at 24 h could confound interpretation of p53 transcriptional activity, we measured gene expression after 6 h of treatment with 1 μM navtemadlin—a concentration at which off-target effects are more likely to be detected. Following navtemadlin treatment, *TP53* mRNA levels were 77% lower in *TP53*-knockdown cells compared to control-knockdown cells (Fig. 2A; *P*_*adj*_ = 0.007); no significant changes were measured in DMSO-treated cells. Similarly, upon navtemadlin treatment, the mRNA levels of canonical p53 targets *CDKN1A* and *MDM2* were >70% lower in *TP53*-knockdown cells than in control-knockdown cells (Fig. 2B, C; *P*_*adj* for *CDKN1A*_ = 0.01; *P*_*adj* for *MDM2*_ = 0.006). Despite the partial knockdown, we observed a rescue of phenotypic cell rounding at 1 μM navtemadlin, consistent with reduced cell death triggered by p53 activation (Fig. 2D).

The blunted activity of navtemadlin in *TP53*-knockdown cells was also evident at the protein level, as measured by western blotting and immunofluorescence staining. In western analysis of lysates treated for 24 h with 0.05 μM navtemadlin, the expression of p53 and p21 were reduced by more than 1.3-fold in *TP53*-knockdown cells compared to control-knockdown cells. At 1 μM navtemadlin, the expression of p53 and p21 were reduced by more than 1.7-fold in *TP53*-knockdown cells, while expression of PUMA was 2.4-fold lower than in control-knockdown cells (Fig. 2E). In immunofluorescence staining, a 44% reduction in p53 and a 35% reduction in p21 levels were also measured in *TP53*-knockdown cells, but only at 1 μM navtemadlin (Fig. 2F–H; *P*_*adj* for p53_ = 0.005; *P*_*adj* for p21_ = 0.02). When taken together, these results provide strong evidence that the molecular and phenotypic effects exerted by navtemadlin are mediated through p53.

### Different protein networks are affected by increasing levels of p53 activation in HUVEC

**Fig. 3:**
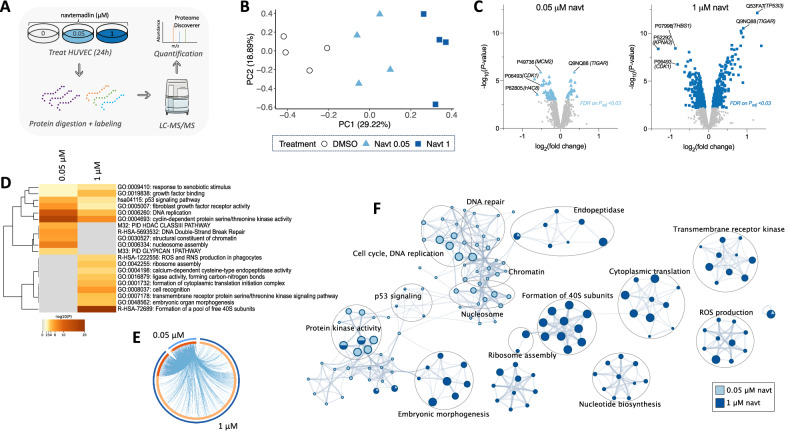
Low levels of p53 activation alter protein networks in DNA replication while high levels alter those involved in stress responses and ribosome assembly. **A** Schematic of proteomics workflow in which HUVEC were treated with navtemadlin for 24 h, digested, and quantified using TMT-based mass spectrometry. **B** Protein abundances (mean-normalized) separate by treatment (DMSO vs navtemadlin), as assessed by principal component analysis. Percentage of variance is shown in parentheses. **C** Volcano plots showing differentially expressed proteins with decreased and increased abundance between DMSO-treated and navtemadlin-treated samples. Downregulated proteins include CDK1 (involved in cell cycle), THBS1 and PECAM (associated with angiogenesis), and KPNA2 (important for nuclear export), while upregulated proteins include those TIGAR and TP53I3 (involved in p53 signaling and apoptosis). *FDR* on *P*_*adj*_ < 0.03. **D** Heat map depicting the top 20 enriched terms within ontological biological processes following p53 activation by navtemadlin. Enriched terms are involved in p53 signaling, DNA replication, and ribosome assembly. Proteins were mapped to their corresponding gene symbols in Metascape for functional enrichment analysis using various ontologies (GO biological processes, GO molecular functions, KEGG pathways, Reactome, and canonical pathways). The color gradient represents *P*-values (as -log_10_). **E** Circos plot depict partial overlap in ontology terms between low and high levels of p53 activation induced by navtemadlin. Dark orange segments indicate shared ontology terms between treatment groups, while light orange segments indicate ontology terms unique to each treatment. Proteins were mapped to their corresponding gene symbols for enrichment analysis using Metascape. **F** Different biological functions are affected at low and high levels of p53 activation induced by navtemadlin. After protein hits were converted to their respective gene symbols (using Metascape), functional analysis was performed to identify enrichment in ontology terms. The top 20 clusters of enriched ontology terms were then selected using a *P*-value < 0.01, a minimum count of 3 genes, and an enrichment factor > 1.5 (i.e., pathway is represented 1.5 more frequently in list than would be expected by chance). Each node (circle) in the network represents an ontology term comprising a group of genes that share a common biological function. The size of the node indicates the number of genes comprising that group, and the color of the pie charts within each node represents treatment groups. Nodes are grouped into clusters using the Kappa similarity score, so that clustered nodes are functionally related. Each cluster is labeled with a representative term summarizing its main biological function.

To identify broader molecular changes associated with graded levels of p53 activation, we performed mass spectrometry-based proteomics on HUVEC lysates treated for 24 h with 0 µM, 0.05 µM, or 1 µM navtemadlin (Fig. 3A). After confirming similarities in the distributions of protein abundance across samples (SI Fig. 1A), we selected mean-normalized data based on dendrogram separation (SI Fig. 1B) to perform principal component analysis. Samples primarily clustered by treatment group (Fig. 3B). Of the 2800 total detected proteins, 87 total proteins at 0.05 µM and 632 total proteins at 1 µM were differentially abundant between the DMSO-treated and navtemadlin-treated samples (two-way ANOVA, FDR < 0.03, SI Fig. 2A). For example, treatment with 0.05 µM navtemadlin resulted in downregulation of proteins involved in cell cycle regulation, such as MCM2, CDK1, and H4C8. In contrast, treatment with 1 µM led to downregulation of many proteins, including THBS1 and PECAM (two proteins involved in angiogenesis), as well as, upregulation of proteins, such as TP53I3 (a p53-induced protein involved in oxidative stress response). Treatment at both concentrations resulted in differential expression of 79 proteins (SI Fig. 2A), including upregulation of proteins such as TIGAR (p53-induced protein involved in apoptosis) and downregulation of proteins such as KPNA2 (a nuclear export protein) (Fig. 3C; SI Fig. 2B–D). Unlike in the navtemadlin-treated tumor cell proteome [24], we did not detect MDM2 or p21 in the navtemadlin-treated endothelial proteome by mass spectrometry; the proteins were, however, detected by western analysis (Fig. 1E). The proteomics results were confirmed by immunoblotting: the expression of THBS1, PECAM and KPNA2 decreased with navtemadlin treatment, while that of TP53I3 and TIGAR increased with treatment (SI Fig. 2E). Given that THBS1 and PECAM are proteins involved in angiogenesis, we also tested whether the decrease measured upon navtemadlin treatment was p53-dependent and confirmed that their expression was rescued in *TP53*-knockdown cells (SI Fig. 2F).

Functional overrepresentation and network analyses were then applied to identify the biological processes enriched within the differentially expressed proteins (Fig. 3D–F). Enriched proteins at the low concentration were associated with Gene Ontology (GO) terms of Biological Processes such as DNA replication (*q*-value = 6.3 × 10^−^^10^) and cell cycle (*q*-value = 2.5 × 10^−^^5^), while those at the high concentration were associated with cytoplasmic translation (*q*-value = 1 × 10^−^^19^) and ribosome biogenesis (*q*-value = 1.6 × 10^−^^4^). By analyzing the network of protein-protein interactions, we observed that dense clusters of proteins within these pathways were affected by navtemadlin treatment (SI Fig. 2B–D). Given that TMT-based MS captures only a subset of the proteome, we ran functional overrepresentation analysis on all the detected proteins to identify which protein families are generally enriched in the endothelial proteome. Among the 52 significant pathways (*P* < 0.05) were those involved in cytoskeletal regulation, integrin signaling, fibroblast growth factor (FGF) signaling, metabolism, cell cycle, and angiogenesis. Together, these results corroborate our cellular assays showing that navtemadlin induces different effects in a concentration-dependent manner: while both low and high concentrations of navtemadlin reduce DNA replication and cell cycle proteins, high concentrations of navtemadlin also affect translation and ribosome biogenesis.

### All tested levels of p53 activation reduce vessel growth and integrity in vitro and in vivo

**Fig. 4:**
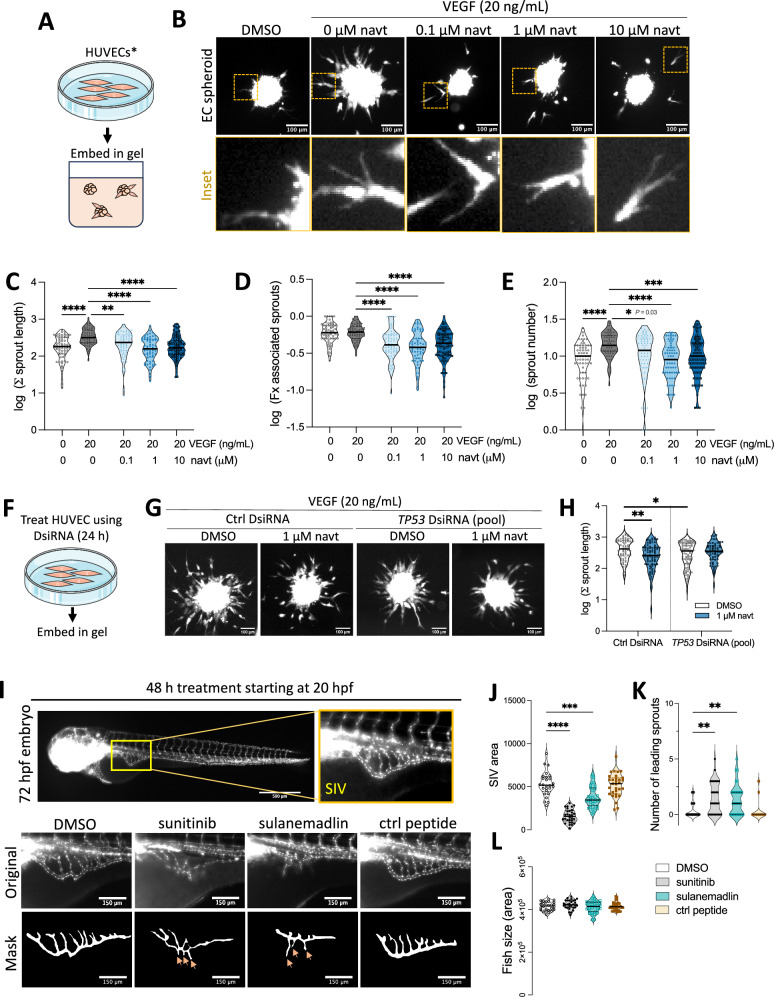
Pharmacological activation of p53 reduces growth of venous vessels in vitro and in vivo at all tested concentrations. **A** Schematic of the endothelial spheroid sprouting assay. HUVEC were embedded in a fibrin gel matrix and treated for 24 h using solvent (DMSO) or increasing concentrations of navtemadlin (navt). Vascular endothelial growth factor (VEGF, 20 ng/mL) was used to stimulate angiogenic sprouting. **B** Fluorescence microscopy images show reduced sprouting in HUVEC spheroids treated with increasing navtemadlin concentrations; the images are brightness and contrast adjusted for clarity. Insets show cell filopodia in sprouts. Scale bars = 100 µm. **C**–**E** All tested concentrations of navtemadlin reduce (**C**) total sprout length, (**D**) fraction of sprouts associated with spheroid body (a measure of connectivity), and (**E**) total sprout number of HUVEC spheroids. Each data point in violin plot indicates one spheroid (*n* = 64 spheroids for baseline; 61 spheroids for VEGF; 66 spheroids for 0.1 and 1 μM navt; 84 spheroids for 10 μM navt; pooled from three experiments). *P_*adj*_ < 0.05, ** *P*_*adj*_ < 0.01, ***P_*adj*_ < 0.001, **** *P*_*adj*_ < 0.0001 using Kruskal-Wallis with Dunn’s correction. Note y-axis is shown on log scale. **F** Schematic of endothelial sprouting assay following 24 h transfection with control or *TP53*-targeting DsiRNAs (pool). After embedding, spheroids were treated for 24 h using DMSO or navtemadlin in the presence of VEGF. **G** Fluorescence microscopy images show that navtemadlin does not further reduce sprouting of HUVEC spheroids after *TP53*-knockdown; the images are brightness and contrast adjusted for clarity. Scale bars = 100 µm. **H** Quantification of total sprout length in drug-treated HUVEC spheroids following transfection with control or *TP53*-targeting DsiRNAs (pooled). Each data point in violin plot indicates one spheroid (*n* = 66 spheroids for DMSO and 82 spheroids for 1 μM navt in Ctrl DsiRNA; 90 spheroids for DMSO and 65 spheroids for 1 μM navt in *TP53* DsiRNA spheroids/group pooled from three experiments). *P_*adj*_ < 0.05, ** *P*_*adj*_ < 0.01, using Kruskal-Wallis with Dunn’s correction. Note y-axis is shown on log scale. **I** A zebrafish embryo (*Danio rerio*) model expressing fluorescent vasculature (*Tg*(fli1:eGFP)) was used to measure effects of p53 activation in developing subintestinal vessels (SIV) in vivo. Embryos were treated with vehicle (DMSO), sunitinib (positive control), sulanemadlin (stapled peptide activator of p53), or control peptide for 48 h, starting at 20 h post-fertilization (hpf). Cropped maximum intensity projections of the subintestinal vessels and corresponding vessel masks (binary images below) are shown; the images are brightness and contrast adjusted for clarity. Arrows (orange) on the binary masks indicate ectopic sprouts. Scale bar of cropped images = 150 µm. **J–L** Treatment with p53 activator sulanemadlin leads to (**J**) reduction in subintestinal vessel area, (**K**) an increase in number of endothelial cell tip extensions, and (**J**) no measurable change in fish size. Each data point in violin plot represents one embryo (*n* = 26 embryos for DMSO; 30 embryos for sunitinib; 32 embryos for sulanemadlin; 31 embryos for control peptide; pooled from two independent experiments). ***P_*adj*_ < 0.001, ****P_*adj*_ < 0.0001 for vessel areas using Brown-Forsythe ANOVA with Dunnett’s T3 correction and *P_*adj*_ < 0.05, **P_*adj*_ < 0.01 for sprout number using Kruskal-Wallis non-parametric test with Dunn’s correction for multiple testing.

Given that disruption of cell cycle progression has previously been shown to increase sprouting and to impair vascular sprout formation, we predicted based on our in vitro and proteomics data that different levels of p53 activation would likely have a similar effect on vessel growth. To test this hypothesis, we used an in vitro sprouting angiogenesis assay (Fig. 4A) in the first instance to measure the sprouting of endothelial spheroids upon exposure to vascular endothelial growth factor (VEGF) and increasing concentrations of navtemadlin (Fig. 4B). Unexpectedly, all tested concentrations of navtemadlin decreased the length of VEGF-induced sprouting (baseline-corrected) in HUVEC spheroids by > 67% (Fig. 4C) and in microvascular HDMEC spheroids by > 80% (SI Fig. 3A, B). In HUVEC spheroids, the fraction of sprouts that were still associated with the spheroid body also decreased (Fig. 4D), as did the total number of sprouts emerging from each spheroid (Fig. 4E). To confirm that the reduced sprouting induced by navtemadlin is p53-dependent, we then performed the sprouting assay in *TP53*- or control-knockdown cells (Fig. 4F). Although *TP53*-knockdown alone reduced sprouting compared to the control knockdown (*P*_*adj* for *TP53* KD vs control KD_ = 0.02), treatment with navtemadlin did not further reduce sprouting in *TP53*-knockdown spheroids (Fig. 4G, H; *P*_*adj* for *TP53* KD DMSO vs *TP53* KD navt_ = 0.48).

Given that migrated sprouts in navtemadlin-treated spheroids still appeared to extend filopodia (Fig. 4B), we subsequently assessed whether navtemadlin treatment led to reduced cell migration using scratch-wound assays on monolayer HUVEC. While VEGF (20 ng/mL) significantly increased migration of HUVEC at 10 h (*P*_*adj*_ = 0.0003), navtemadlin did not significantly reduce VEGF-induced migration at either of the tested concentrations (SI Fig. 4A, B); further time points were not measured to avoid confounding effects of cell proliferation. We confirmed that navtemadlin treatment for 10 h induced p53 and p21 levels using both western blotting and immunofluorescence staining, excluding the possibility that the drug had no effect at this time point (SI Fig. 4C, D).

Because sprout stability is typically maintained by stalk cells through formation of tight and adherent junctions in the growing vessel [25], we reasoned that navtemadlin may affect the connectivity between endothelial cells. Using immunofluorescence staining and an endothelial permeability assay on confluent endothelial monolayers, we found that both concentrations of treatment led to a reduction in the expression of zona occludens-1 (a tight junction marker) and VE-cadherin (an adherent junction marker; SI Fig. 5A) after 24 h. Additionally, both concentrations also increased the permeability (or leakiness) of a fluorescent dextran across the endothelial barrier (SI Fig. 5B, C). Although the increased permeability measured at 1 µM navtemadlin may have resulted from an increase in cell death (SI Fig. 5D–F), the increased permeability measured at 0.05 µM navtemadlin without significant cell death suggests that even low levels of p53 activation may affect vessel integrity. When combined, these in vitro results indicate that all tested levels of p53 activation impair vessel growth and integrity, although effects at the higher concentration may be confounded by cell death.

To determine whether these in vitro results using venous endothelial cells translated in vivo, we then measured the growth of angiogenic venous vessels in zebrafish embryos [9] treated with sulanemadlin (Fig. 4I). This stapled peptide was used in vivo because we previously found that it had higher in vivo efficacy than small molecule MDM2 inhibitors [26]. By imaging a transgenic zebrafish model in which endothelial cells are fluorescently labelled (*Tg*(fli1:eGFP)), we found that the growth of the vessels in the subintestinal venous plexus was reduced by > 30% following 48 h treatment of sulanemadlin or sunitinib (positive control) compared to solvent-treated controls (Fig. 4J). In contrast, no significant changes were measured following treatment with the control stapled peptide (Fig. 4J). Previous studies showed that ectopic sprouts form in the subintestinal vessels prior to vessel elongation and subsequent retraction during vessel maturation [9]. In embryos treated with sulanemadlin or sunitinib, these leading sprouts remained during treatment, instead of retracting into the vessel basket (Fig. 4I, K). Reductions in the growth of subintestinal vessels could not be explained by abnormal growth, as none of the treatments resulted in a significant change in fish size (Fig. 4L). When combined, the results from the in vitro and in vivo sprouting assays indicate that p53 activation reduces the growth of venous vessels.

### Increasing levels of p53 activation induce different effects in tip-like and non-tip-like cells

**Fig. 5:**
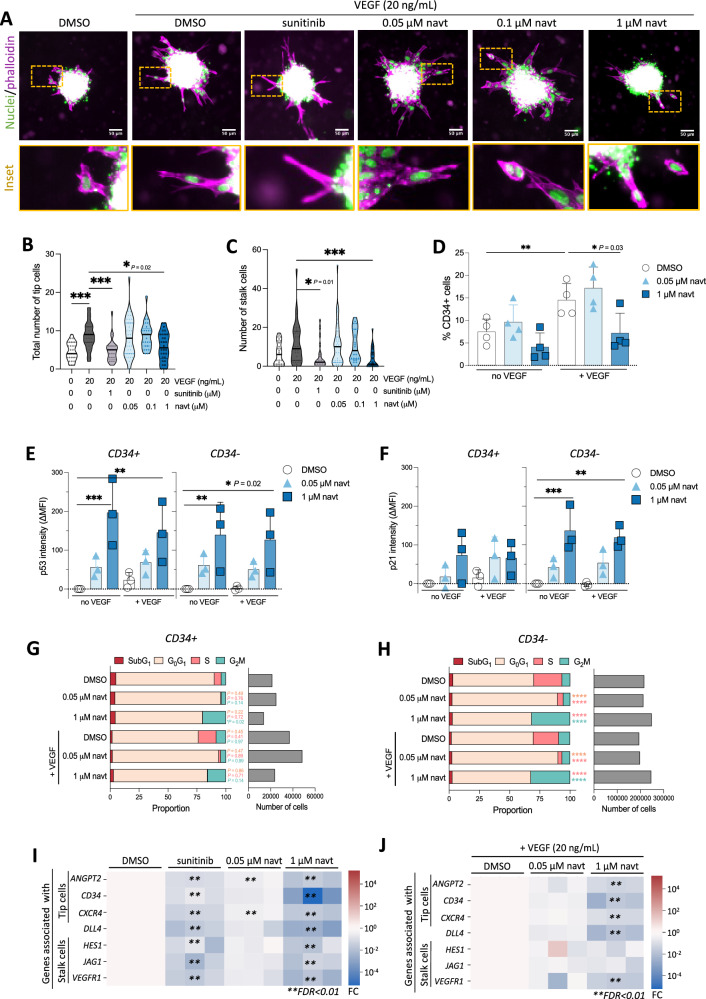
p53 activation differentially modulates cell fate in tip-like and non-tip-like cells. **A** Epifluorescence microscopy images show altered sprout morphology of HUVEC spheroids treated using sunitinib or increasing concentrations of the p53 activator navtemadlin (navt; 0.05 µM, 0.1 µM, 1 µM) in the presence of VEGF (20 ng/mL). DMSO was used as solvent control. Spheroids were stained for nuclei (green pseudo-color, Hoechst) and sprouts (magenta pseudo-color, phalloidin). Scale bar: 100 µm. **B**, **C** Reduction in the total number of (B) tip cells and (C) stalk cells quantified per spheroid upon treatment with sunitinib or high concentrations of navtemadlin (1 µM) in the presence of VEGF (20 ng/mL). Each symbol in violin plot represents one spheroid (*n* = 30 spheroids for baseline; 31 spheroids for VEGF; 25 spheroids for sunitinib; 38 spheroids for 0.05 μM navt; 25 spheroids for 0.1 μM navt; 30 spheroids for 1 μM navt; pooled from two independent experiments). ***P_*adj*_ < 0.001, ****P_*adj*_ < 0.0001 using Kruskal-Wallis with Dunn’s correction. **D** Treatment with high concentrations of navtemadlin (1 μM) reduces the VEGF-induced increase in the percentage of CD34+ (tip-like) cells, as measured by flow cytometry analysis of treated HUVEC monolayers. Each data point represents value from one experiment (*n* = 4 experiments). Bar height indicates mean value ± SD. ***P_*adj*_ < 0.001, ****P_*adj*_ < 0.0001 using one-way ANOVA with Dunnett’s correction. **E**, **F** Activation of p53 by high concentrations of navtemadlin (1 μM) induces significant p21 activity in CD34− (non-tip-like) cells. The change in the median fluorescence intensity of (**E**) p53 and (**F**) p21 were measured by flow cytometry in CD34+ (tip-like) and CD34− cells following 24 h treatment of HUVEC monolayers in the absence and presence of navtemadlin and VEGF (20 ng/mL). Each data point represents value from one experiment (*n* = 3 experiments). Bar height indicates mean value ± SD. ***P_*adj*_ < 0.001, ****P_*adj*_ < 0.0001 using two-way ANOVA with Dunnett’s correction. **G**, **H** Navtemadlin treatment significantly alters cell cycle distribution of CD34− cells at low concentrations (0.05 μM), and of both CD34+ and CD34− cells at high concentrations (1 μM). The proportion of cells in the different phases of the cell cycle (SubG_1_, G_1_, S, G_2_/M), as well as the total number of cells analyzed in each treatment, were measured using flow cytometry. Bar graphs represent summed values from all experiments (*n* = 4 experiments). ***P_*adj*_ < 0.001, ****P_*adj*_ < 0.0001 using two-way ANOVA with Dunnett’s T3 correction. **I**, **J** In HUVEC monolayers, treatment with navtemadlin (1 µM) primarily alters the expression of genes associated with tip cells. Heatmaps depict fold changes (FC) in mRNA expression of tip cell genes (*ANGPT2*, *CD34*, *CXCR4*, *DLL4*) and stalk cell genes (*HES1*, *JAG1*, *FLT1*) following 24 h treatment with sunitinib or navtemadlin, in the absence or presence of VEGF (20 ng/mL). Expression is normalized to DMSO controls and analyzed using NORMA-Gene [50]. Each square represents the averaged value from one experiment (*n* = 3 experiments). ** *FDR* < 0.01.

We hypothesized that reduced vessel growth could result from decreased proliferation of stalk cells upon p53 activation within growing vessels. Indeed, p53 can be stabilized more readily in proliferating tissues than in quiescent tissues, likely reflecting its role in regulating the maturation and genomic integrity of fast-renewing cells [27]. To test this hypothesis, we first measured whether p53 activation reduced the formation of tip (i.e., leader) or stalk (i.e., follower) cells (Fig. 5A). In HUVEC spheroids, treatment with low concentrations of navtemadlin had no significant effect on tip or stalk cell numbers, while high concentrations significantly reduced the number of tip cells and the number of stalk cells by > 33% to 45%, respectively (Fig. 5B, C).

Because the reduction in formation of tip cells was unexpected, we subsequently delineated p53 activity and cell cycle changes in each population using flow cytometry detection of CD34, a molecular marker expressed by cultured endothelial cells that exhibit tip-like features [28, 29] (SI Figs. 6, 7). While low concentrations of navtemadlin had no measurable effect, high concentrations of navtemadlin reduced the frequency of CD34+ (tip-like) cell formation in the presence of VEGF (Fig. 5D), corroborating the results observed in spheroids. To assess whether this reduction was due to increased p53 activity, we measured the levels of p53 and that of p21 (as a marker for p53 transcriptional activity) in CD34+ (tip-like) and CD34− (non-tip-like) cells. After navtemadlin treatment, p53 levels increased in both CD34+ and CD34− cells in the absence (*P*_*adj for CD34+ cells*_ = 0.007; *P*_*adj for CD34- cells*_ = 0.02) and presence of VEGF (*P*_*adj for CD34+ cells*_ = 0.0003; *P*_*adj for CD34- cells*_ = 0.009) (Fig. 5E). In contrast, significant increases in p21 levels were measured only in CD34− cells in the absence (*P*_*adj for CD34+ cells*_ = 0.09; *P*_*adj for CD34- cells*_ = 0.0006) and presence of VEGF (*P*_*adj for CD34+ cells*_ = 0.14; *P*_*adj for CD34- cells*_ = 0.003) (Fig. 5F). However, it is possible that changes in CD34+ could not be detected with sufficient statistical power due to the lower abundance of this population.

Given that p21 induction was significantly increased in CD34− cells, we subsequently assessed whether p53 might differentially affect their cell cycle distribution. While p53 activation had no significant effect on the proliferation or G_0_G_1_ phases of CD34+ cells (Fig. 5G), it altered the cell cycle phases of CD34− cells depending on the level of p53 activation (Fig. 5H). At low concentrations, it reduced the proliferation of CD34− cells and increased G_0_G_1_ arrest, but at high concentrations, it reduced proliferation and increased G_2_M arrest. At high concentrations, p53 activation also significantly increased G_2_M arrest in CD34+ cells. Thus, although cell cycle changes were strongly detected in CD34− cells, we cannot exclude the possibility that additional effects occurred in CD34+ cells but were not detected due to lower abundance.

Based on these data, we reasoned that p53 activation in CD34+ cells may affect their formation through mechanisms other than cell cycle regulation. One possibility is that p53 activation leads to reductions in the expression of key tip cell genes needed for tip cell formation. Using RT-qPCR, we found that treatment with low concentrations of navtemadlin alone reduced expression of two tip cell genes *ANGPT2* and *CXCR4*, while treatment with high concentrations reduced the expression of all tip and stalk cell genes (Fig. 5I). However, in the presence of VEGF, p53 activation reduced all the tip cell genes but only one of the stalk cell genes (Fig. 5J). Taken together, these results suggest that p53 activation influences the cell fate of tip-like and non-tip-like cells in a concentration-dependent manner: low levels primarily affect proliferation in non-tip-like cells, while high levels impair both cell cycle regulation and angiogenic gene expression in both populations.

## Discussion

### Effects of pharmacological p53 activation on distinct cell fates in endothelial cells

In this study, we evaluated whether different levels of p53 could modulate endothelial cell fate by triggering distinct cellular responses during angiogenesis. By measuring the molecular and phenotypic effects induced by low and high levels of p53 activation, we found that increasing p53 levels transitioned endothelial cells from cell cycle arrest to cell death. Notably, increasing levels of p53 also triggered distinct effects in tip-like and non-tip-like cells. At low p53 levels, migrating tip-like cells were not measurably affected, while proliferating non-tip-like cells were arrested. However, at high levels of p53 activation, both populations were affected, although some effects in tip-like cells may not have been fully captured due to their lower abundance. Despite these distinct effects on cell fate, all tested levels of p53 activation surprisingly resulted in reduced vessel growth in vitro and in vivo. These findings suggest that the level of p53 activation could be a novel way to selectively modulate endothelial cell fate while still disrupting abnormal vessel growth in diseases such as cancer and ischemic conditions.

An important finding in our study is that activation of p53 induced cell cycle arrest or cell death in a concentration-dependent manner, as evidenced by our in vitro molecular experiments and proteomics analysis. The graded effects of p53 activation on endothelial cell fate are consistent with the threshold model of p53 activation [16, 30] and with results from previous studies in other cell types [16]. Notably, we found that the effects of p53 activation at lower concentrations could be recovered and induced primarily in proteins involved in cell cycle regulation. These results suggest a possible therapeutic window wherein endothelial function could be modulated without inducing cellular damage. In contrast to the effects induced by low levels of p53 activation, the effects of p53 activation at higher concentrations led to a stressed phenotype, as evidenced by alterations in proteins involved in ribosome assembly and oxidative stress. Interestingly, the proteins most upregulated in our proteomics data, TIGAR and TP53I3, have previously been shown to play a role in p53-induced apoptosis by regulating oxidative stress [31, 32], and their overexpression in endothelial cells has been shown to reduce angiogenesis [33]. Together, our data provide new insights into how graded levels of p53 activation affect endothelial cell fate.

Although our study used well-characterized new generation p53 activators (navtemadlin and sulanemadlin), off-target effects remain a concern in pharmacological studies. We confirmed that the effects of 1 μM navtemadlin on cell fate in HUVEC are largely p53-dependent, as *TP53*-knockdown rescued the molecular and phenotypic outcomes in transcriptional, protein, and sprouting assays. We have previously shown that similar high concentrations of navtemadlin (1.5 µM) had negligible effects on the proteome of p53-null tumor cells, further supporting that the observed effects are primarily on-target [24]. By using a pharmacological approach to evaluate the graded effects of p53 activation on cell fate, we demonstrate that the cellular fate of endothelial cells depends on the level of p53 activation. This novel finding unifies previous conflicting reports that p53 activation in endothelial cells induces either cell cycle arrest [13] or apoptosis [14].

### Reduction of vessel growth despite distinct effects on cellular fate

Unexpectedly, despite the distinct cellular fates induced by graded levels of p53 activation, overall vessel growth was reduced at all tested concentrations in both in vitro and in vivo assays. How can these different cellular fates lead to an overall reduction in vessel growth? One explanation is that stalk cell arrest at low levels of p53 activation disrupts the proliferation and connectivity of the nascent vessel, with little impact on tip cell migration and/or specification. Our data from immunofluorescence staining of tight and adherens junctions, permeability assays, flow cytometry experiments, and ectopic sprout formation in subintestinal vessels of zebrafish embryos are consistent with this explanation. However, previous studies using the p53 activator roscovitine showed that forced cell cycle arrest promotes excessive sprouting [7]. Although we did not observe enhanced sprouting in our experiments, we cannot rule out such a compensatory mechanism because it may not have been detectable using our analysis methods or the concentrations used in this study. Time-resolved imaging at even lower concentrations in future studies could help clarify this possibility.

In contrast to the low levels of p53 activation, we found that the reduction in vessel growth at high p53 activation levels likely results from loss of both tip-like and non-tip-like cell populations, possibly due to induction of oxidative stress and cell death mechanisms. Although previous studies have shown that p53 activation in endothelial cells reduces vessel growth by inducing cell death, our study is the first to show that the high levels of p53 alter the frequency of tip-like cells, as well as the cell cycle phases and angiogenic gene expression of both cell populations. By demonstrating the distinct effects of graded p53 levels on endothelial cell phenotypes, our study offers new insights into the ways in which p53 regulates angiogenesis.

### Implications for p53’s role in angiogenesis

We found that venous endothelial cells are remarkably sensitive to p53 activation both in vitro and in vivo. In vitro, p53-treated venous endothelial cells stopped growing at an entire log-fold concentration lower than in other primary cell types, such as primary fibroblasts. In vivo, venous-derived blood vessels had reduced growth after p53-treatment. Given that vessel growth was reduced without any observed effect on fish size, and that p53 activation is embryonically lethal in *mdm2* knockout zebrafish [34], these findings suggest that these vessels may have a lower threshold of sensitivity to p53 activation than other cell types. The increased susceptibility of venous endothelial cells may stem from transcriptional differences, as venous endothelial cells express higher levels of cell cycle genes than other types of endothelial cells [4]. These findings have important implications. First, while fibroblasts are commonly used as normal cells in p53 activation studies [17, 24], our results underscore the need to include endothelial cells in such studies to understand the effects of p53 activators on normal cells. Second, the sensitivity of venous endothelial cells could have implications for diseases where venous vessels are predominantly affected, such as varicose veins or venous thrombosis [35]. Future studies in diseased conditions and disease-relevant models are needed to build on the insights from the in vitro and zebrafish embryo models.

In conclusion, our study advances the understanding of p53’s role on endothelial cell phenotypes during vessel growth. We found that graded levels of p53 activation induce distinct cell fates in normal endothelial cells by affecting protein networks involved in DNA replication or ribosome biogenesis. Intriguingly, all tested levels of p53 activation reduced vessel growth, possibly because graded levels of p53 may exert distinct effects on tip-like and non-tip-like cell populations. Our findings provide novel insights into how p53 regulates angiogenesis, which could be used to refine vascular-targeted therapies. Further research is needed to explore how p53 modulation could be leveraged to target dysfunctional tip and stalk cell development in vascular diseases, such as cancer and age-related macular degeneration.

## Materials and methods

### In vitro experiments

#### Chemicals and proteins

To pharmacologically activate p53, we used nutlin-3a and navtemadlin (two small molecule MDM2 inhibitors) [17–19], sulanemadlin (a stapled peptide MDM2/MDMX inhibitor) [20]; a control stapled peptide was used as a negative control with minimal activity. Sunitinib malate (a potent inhibitor of tyrosine kinases including vascular growth factor receptor-2 [36]) was used as a positive control to inhibit angiogenesis. Sulanemadlin was synthesized (DeliverTides LLC) with sequence Ac-Leu-Thr-Phe-R8-Glu-Tyr-Trp-Ala- Gln-Leu-S5-Ala-Ala-Ala-Ala-Ala-DAla- NH_2_ (olefin staple from R8 to S5), while the control peptide was synthesized with the same sequence except replaced with the D-isomer of phenylalanine [26]. To stimulate sprouting, we used 20 ng/mL vascular endothelial growth factor (VEGF_165_, cat # 11458-CE, R & D systems). All compounds were dissolved in DMSO to the following final concentrations: 40 mM nutlin-3a (Sigma Aldrich), 40 mM navtemadlin (Axon MedChem), 51.2 mM sulanemadlin (custom-made), 40 mM control stapled peptide (custom-made), and 50 mM sunitinib malate (Sigma Aldrich). VEGF was dissolved in phosphate-buffered saline (PBS) containing 0.1% bovine serum albumin at 100 mg/mL. Stock solutions were stored in 20 µL aliquots at −20 °C and were diluted to lower concentrations for experiments.

#### Cell culture

For in vitro assays, we used commercially available primary cultures of three cell lines: human umbilical vein endothelial cells (HUVEC) from pooled donors (cat # C-12203, Promocell), human dermal microvascular endothelial cells (HDMEC) from adult donors (cat # C-12212, Promocell), and normal human dermal fibroblasts (NHDF) from adult donors (cat # 106-05 A, Cell Applications Inc). HUVEC were cultured in Endothelial Cell Growth Medium-2 Kit (cat # C-22011, Promocell) supplemented with all recommended growth factors and serum. HDMEC were cultured in Endothelial Cell Growth Medium MV2 (cat # C-22022, Promocell) supplemented with all recommended growth factors and serum. NHDF were cultured in Fibroblast Growth Medium 2 (cat # C-23020, Promocell) supplemented with all recommended growth factors and serum. Cells were passaged using DetachKit2 (cat # C-41202, Promocell). All cells were maintained in a humidified incubator (37 °C, 21% O_2_, 5% CO_2_) up to passage 5 and were regularly checked for the absence of mycoplasma (Lonza MycoAlert™, Lonza).

#### Treatment of HUVEC using Dicer-substrate siRNA (DsiRNA)

To determine whether the molecular and phenotypic changes induced by navtemadlin were p53-dependent, we used Dicer-substrate small interfering RNA to knockdown *TP53* in HUVEC. Predesigned DsiRNAs against *TP53* (hs.Ri.TP53.13.1, hs.Ri.TP53.13.2, hs.Ri.TP53.13.3), a control DsiRNA that targets a non-human sequence, a positive control DsiRNA targeting *HPRT1*, and a fluorescently labeled TYE-563 DsiRNA to assess transfection efficiency were purchased as part of the TriFECTa RNAi Kit (hs.Ri.TP53.13, Integrated DNA Technologies). The DsiRNAs were reconstituted in nuclease-free water, diluted in duplex buffer, and stored in aliquots at −20 °C as per kit instructions. To transfect HUVEC, complexes comprising a 1:1 mixture of DsiRNA:lipids were prepared in low-serum Opti-MEM (cat#11058021, Gibco) by incubating DsiRNAs (10 nM per duplex) with Lipofectamine RNAiMAX (0.6 μL per 75 μL, cat#13778030, Thermo Fisher) for 20 min at RT, and then added to cells incubated in an equivalent volume of EGM2 complete medium. For all knockdown experiments, the three DsiRNAs targeting *TP53* were pooled. Transfection efficiency was assessed by fluorescence of a TYE-563 DsiRNA in Incucyte® SX5 (Sartorius) at 24 h post-transfection, while knockdown efficiency was measured by RT-qPCR of *HPRT1*. The medium was then replaced with fresh EGM2 complete medium containing the respective drug treatments for downstream assays.

#### Monolayer growth assays

The monolayer growth of primary human cells was measured upon pharmacological activation of p53. Cells were seeded (3 × 10^3^ cells/well) into a 96-well plate (Techno Plastic Products AG), allowed to attach for 24 h, and then treated with a range of drug concentrations diluted in their respective medium. Cells were imaged in real-time (Incucyte® S3) for a total of 72 h after treatment. For growth reversibility experiments, cells were treated with drug-containing medium for 24 h, washed using fresh medium, and then incubated in fresh medium without drugs for an additional 48 h of imaging. Cell confluence was normalized to the untreated control cells for growth assays and to that at the starting time point for reversibility experiments. The concentrations of inhibitor that reduced growth by 50% (IC_50_) were calculated using a non-linear standard slope model (inhibitor vs response—three parameters; Graph Pad Prism 9.0).

#### Western blotting

We used western blotting to assess the expression of downstream targets induced by p53 activation and to verify some of the top hits from our proteomics data. HUVEC (4 × 10^5^ cells/dish) were seeded in 60 mm dishes (3 mL/dish), allowed to attach for 36 h, treated with drugs for 10 h or 24 h, and lysed using SDS buffer as previously described [37]. For knockdown assays, HUVEC (2×10^5^ cells/dish) were treated with siRNA for 24 h, then treated with fresh medium containing drugs for 24 h, and lysed. Electrophoresis and immunoblotting were performed [37] using various antibodies to detect the following proteins: p53 [38], MDM2, p21, PUMA, TIGAR, TP53I3, KPNA2, PECAM, and THBS1 (see Supplementary Table 1 for antibody information and concentrations). The blocking solution for primary antibodies was 5% milk in TBS-T, except for p21 for which the blocking solution was 5% bovine serum albumin in TBS-T. Primary antibodies were detected using the following horseradish peroxidase (HRP)-conjugated secondary antibodies: swine anti-rabbit HRP (1:2000, cat # P0217, Dako) or goat anti-mouse HRP (1:1000, cat # P0447, Dako). Protein loading was assessed using stain-free imaging (BioRad), while protein expression was detected by chemiluminescent imaging using ECL Clarity Substrate and Reagent (cat # 1705060, BioRad) on a ChemiDoc^TM^ Touch Imaging System (cat # 1708370, BioRad). Images were cropped from full blots, which are shown in supplementary material (see ‘Full Length Western Blots’).

#### Immunofluorescence staining in monolayers

Immunofluorescence staining was used to detect treatment-induced changes in protein levels of p53, p21 (marker of cell cycle arrest), Ki67 (marker of proliferation), zona occludens-1 (ZO-1, tight junction protein), and vascular endothelial-cadherin (VE-cadherin, protein for endothelial adhesion) in monolayer endothelial cells (see Supplementary Table 1 for antibody information and concentrations). For staining of p53, p21, and Ki67, HUVECs (1 × 10^3^ cells/well) were seeded in 15-well μ-slide angiogenesis plates (cat # 81506 IbiTreat, Ibidi), allowed to attach 36 h, and treated with drugs for 24 h. For staining of p53 and p21 in knockdown assays, HUVECs (1 × 10^3^ cells/well) were seeded in 18-well μ-slide angiogenesis plates (cat # 81816, IbiTreat, Ibidi), allowed to attach 36 h, treated for 24 h with DsiRNA, and then treated with drugs for 24 h. Cells were fixed with 4% formaldehyde for 10 min, and permeabilized and blocked using PBS containing 5% bovine serum albumin (Sigma), 5% goat serum (Dako) and 0.5% Triton X-100 (Sigma). Cells were subsequently incubated with primary antibody overnight at 4 °C in PBS containing 0.5% bovine serum albumin, 2% goat serum, and 0.25% Triton X-100. Following 3 washes in PBS, they were then incubated with secondary antibody for 60 min at RT, washed 3 times in PBS, and stained for nuclei using Hoechst 34422 for 10 min at RT before mounting. For staining of ZO-1 and VE-cadherin, seeded cells (5 × 10^3^ cells/well) were grown to confluent monolayers in 18-well staining plates (cat # 81816 IbiTreat, Ibidi), treated for 24 h, fixed with 4% formaldehyde for 10 min, permeabilized for 5 min using 0.4% Triton X-100 in PBS, and then stained using the above solutions containing no permeabilization agents. Images were acquired using epifluorescence microscopy (10x objective, 0.30 NA, 0.64 μm resolution; lasers 395 nm and 470 nm or 640 nm; Nikon TiE microscope). To quantify the fluorescence intensities of p53, p21, and Ki67, we measured the average intensity of pixels within cell nuclei on median-filtered images using a 2 × 2 kernel size based on modified versions of published analysis [39].

#### Colorimetric staining in monolayers

For staining of β-galactosidase (marker of senescence), colorimetric staining was performed using the Senescence β-galactosidase Staining Kit (9860, Cell Signaling Technologies) according to manufacturer’s instructions. For quantification of colorimetric signals, a background subtraction using rolling ball was applied to all the images before measuring the number of pixels above a user-defined threshold (ImageJ).

#### Live imaging of cell death in monolayers

Induction of cell death by pharmacological p53 activation was measured in real-time using a fluorescence dye that binds nucleic acids in cells that have compromised plasma membranes. For assays involving exponentially growing cells (Fig. 1F), HUVEC were seeded (3×10^3^ cells/well) into a 96-well plate (TPP), allowed to attach for 24 h, and then treated with medium containing 100 nM SytoxGreen (cat # S7020, ThermoFisher) and DMSO, navtemadlin (0.05 µM or 1 µM), or staurosporine (100 nM). Cells were then imaged in real-time (Incucyte® S3) every 6 h using the brightfield and GFP channels (excitation filter 488). For assays involving confluent monolayers (SI Fig. 5), cells were seeded into a 96-well plate (TPP), grown to confluence, treated with medium containing drugs and SytoxGreen, and imaged in real-time (Incucyte® SX5). For both types of assays, the fluorescence intensity was quantified using the total integrated intensity using the Incucyte® Software (Essen Bioscience).

#### Scratch wound assay

To determine whether endothelial cell migration was affected by treatment, we used a scratch wound assay. Briefly, endothelial cells were seeded (3 × 10^4^ cells/well) in a 96-well plate (Essen Bioscience) for 24 h and then incubated in EGM2 basal medium containing 2% fetal bovine serum for 6 h prior to making a scratch using the scratch wound maker (Essen Bioscience); longer incubations in this medium resulted in apoptosis. Cells were then washed once with PBS, treated with drug-containing medium (made in EGM-2 containing all growth supplements and 2% fetal bovine serum), and imaged immediately and at 10 h following scratch using the Incucyte® software (Essen Bioscience). The percent of the original scratch area that was covered by cells at 10 h was quantified for each image. For staining of p53 and p21 in scratch wound wells, HUVEC were seeded and grown to confluence in a black, 96-well, clear bottom plate (Corning 3603) as described above, scratched manually using a pipette, and then fixed at 10 h following the initiation of migration. Staining was performed as described in the *Immunofluorescence staining* section.

#### Monolayer permeability assay

The effect of p53 activation on endothelial monolayer permeability was tested using a Transwell permeability assay. Endothelial cells were seeded (10000 cells/cm^2^) and cultured for five days in Transwell inserts until a confluent monolayer was formed; half the medium was exchanged with fresh medium every 48 h. Cells were then treated with fresh EGM-2 complete medium containing DMSO, thrombin (10 U/mL, cat # T4648, Sigma) as a positive control, and two concentrations of navtemadlin for 24 h. The medium in the insert was then replaced with one containing 0.1 μM FITC-dextran (70 kDa, cat # 46945, Sigma), while the bottom of the insert was replaced with EGM-2 complete medium. After 30 minutes of incubation, 100 μL of the medium from the bottom well was pipetted into a 96-well black plate, and fluorescence intensity was measured at 520 nm (Cytation 5 plate reader).

#### Proteomics sample preparation

Changes in the endothelial proteome after pharmacological p53 activation were measured using mass spectrometry-based proteomics. Endothelial cells were seeded (10000 cells/cm^2^), allowed to attach for 24 h, and treated with drug-containing medium for 24 h. Cells were harvested using the DetachKit2 (Promocell) and washed twice with dPBS. Pellets were snap frozen on dry ice for 10 min and stored at −80 °C until analysis. Samples were prepared and analyzed as described previously [40]. In brief, cell pellets were lysed with 100 µL of 8 M urea, 0.1% ProteaseMAX (Promega) in 100 mM Tris-HCl (pH 8.5) with protease inhibitors. The samples were sonicated and centrifuged at 10,000xg before the supernatant was used for protein quantification via BCA assay. A 25 µg aliquot of protein was reduced with 0.6 µL of 0.5 M dithiothreitol (DTT), alkylated with 1.8 µL of 0.5 M iodoacetamide, and digested with 2.5 µL of 0.5 µg/µL Lys-C for 4 h, followed by tryptic digestion overnight. After cleanup on a C18 Hypersep plate, the peptides were labeled with TMTpro and quenched with hydroxyamine.

#### Data acquisition using liquid chromatography-tandem mass spectrometry

The combined TMTpro labeled peptides of biological replicates were separated on a 50 cm C18 column (Thermo Fisher Scientific) using a gradient of 4–26% solvent B (98% acetonitrile, 0.1% formic acid) over 120 min at 300 nL/min. MS1 spectra were acquired on a Q Exactive HF mass spectrometer with a resolution of 120,000 (at m/z 200), targeting 1 × 10^6^ ions. The top 18 precursor ions were fragmented by higher-energy collisional dissociation, and tandem MS was acquired with 60,000 resolution. Data-dependent analysis used dynamic exclusion for 45 s.

#### Proteomics and overrepresentation analysis

The raw data were analyzed with Proteome Discoverer v2.5 using Mascot against the human protein database. The search allowed up to two missed cleavages and included carbamidomethylation of cysteine as a fixed modification, and oxidation, deamidation, and TMTpro labeling as dynamic modifications. Initial search results were filtered to a 5% false discovery rate (*FDR*) using Percolator node in Proteome Discoverer, and quantification was based on reporter ion intensities.

Raw protein abundance values were normalized and inspected using the NormalyzerDE software [41]. To identify changes induced by treatment, we then performed differential expression analysis using mean normalized values because this normalization method provided adequate data distribution and group separation in dendogram plots. Differential expression analysis was performed in NormalyzerDE using LIMMA empirical Bayes moderated t-statistics [42]. Differential expression data were imported into OmicLoupe [43] to visualize volcano scatterplots and filtered using a *FDR* < 0.03 as the cutoff to obtain a reasonable length of the list of proteins in both treatment groups for the functional pathway analysis. Principal component analysis was performed to assess whether samples could be separated by treatment using the prcomp function in R (v. 2022.02.3 build 492).

To identify functional pathways that were overrepresented in each group (low, shared, and high concentrations), we ran the protein lists (*FDR* < 0.03) in Metascape [44] using a custom background including all 2761 detected proteins in the experiment. The follow terms were used for ontology sources: GO molecular functions, GO biological processes, KEGG pathway, Reactome gene sets, and Canonical Pathways. To identify terms with membership similarities, those with a raw *P*-value < 0.01 (calculated using cumulative hypergeometric distribution), a minimum count of 3, and an enrichment factor >1.5 were grouped into clusters. Multiple testing correction, performed using the Benjamini-Hochberg procedure, was used to calculate *q*-values. Hierarchical clustering of enriched terms was performed using kappa scores as similarity metric and those with similarity >0.3 were clustered as sub-trees. Circos plots were generated using the default settings in Metascape [44].

To check that angiogenesis-related pathways (e.g., VEGF signaling, platelet derived growth factor signaling) could be detected by our proteomics setup, the custom background list was also assessed by overrepresentation analysis using Pathways, Ontologies, and Cell Types on Enrichr [45]. Protein networks were generated using Cytoscape via RCy3 in R [46, 47]. After loading a String network of all detected proteins (taxonID 9606, interaction cutoff of 0.4), we mapped expression data on a force-directed layout using log_2_ fold change values (visualized by color) and negative log_10_ *P*-values (visualized by node size) from the 0.05 μM vs DMSO comparison or the 1 μM vs DMSO comparison. Protein networks were clustered in Cytoscape using the Leiden clustering algorithm (Clustermaker app) with a resolution parameter of 0.5, beta value of 0.01, and 20 iterations. The clusters were applied onto the force-directed layout and manually adjusted to improve visual clarity and group separations.

#### Endothelial spheroid sprouting assay

The effect of p53 activation on sprouting angiogenesis was measured in vitro. HUVEC or HDMEC spheroids were generated and embedded in fibrin gel, as previously described [48]. The spheroids were then treated for 24 h with either DMSO, VEGF (20 ng/mL), or a mixture of VEGF (20 ng/mL) and increasing concentrations of navtemadlin. For sprouting assays in knockdown cells, HUVEC were seeded (1 × 10^5^ cells/well) in 6-well plates and transfected with DsiRNA for 24 h prior to generation and embedding in fibrin gel. Spheroid sprouting was imaged using fluorescence microscopy as previously described and quantified using custom-made software [48] in MATLAB (v. 2024a).

#### Phalloidin staining and quantification in sprouting spheroids

To measure the effect of p53 activation on tip and stalk cell formation, HUVEC spheroids were generated and drug-treated (as described in *Endothelial spheroid sprouting assay* section) for 24 h before being fixed in PBS containing 4% formaldehyde (Sigma Aldrich #28908) for 10 min. Spheroids were stored in PBS at 4 °C with a plastic sealant to minimize evaporation. Staining was initiated by replacing the PBS with a blocking solution composed of 0.2% Triton X-100 and 1% bovine serum albumin in PBS. A total of 100 µL of the blocking solution was added to each well, followed by incubation overnight at RT with gentle shaking (IKA rocker) at 50 rpm. The following day, a staining solution containing 0.16 µM Alexa Fluor™ 647 phalloidin (cat # A22287, Invitrogen) or Alexa Fluor™ 488 phalloidin (cat # A12379, Invitrogen) and 1 µg/mL Hoechst 33342 (cat # H1399, Thermo Fisher) in the blocking solution. Spheroids were then incubated with 50 µL of the staining solution in each well for at least 40 h at RT with shaking. After incubation, the supernatant was removed from each well, and the well was washed three times with Dulbecco’s phosphate-buffered saline (dPBS). To preserve the spheroids for imaging, 200 µL of 80% glycerol in dPBS was added to each well, and the plate was incubated overnight at 4 °C. Prior to imaging, the plate was brought to room temperature for 2 h.

Stained spheroids were imaged using epifluorescence microscopy (10x objective, 0.30 NA, 0.64 µm resolution; Nikon TiE microscope). Excitation wavelengths of 395 nm and 470 nm or 640 nm were used to detect the nuclear and phalloidin staining, respectively. The focus plane was set on 395 nm. At least five spheroids were individually imaged in each treated well, and > 20 spheroids in total were imaged from two independent experiments and pooled for analysis. The NIS Elements software (v 5.02.03) was used to export images (512 μm × 512 μm in x and y, 1.5 MB in nd2 format). The number of tip cells and stalk cells was manually counted using ImageJ (v. 1.52 h, National Institutes of Health). Tip cells were identified as the leader cell of a sprout, while follower cells were identified as those behind the leader cell up until the spheroid body. The counting was manually performed by an investigator who was blinded to the treatment conditions.

#### Flow cytometry

The differential effects of p53 activation on p53 activity and cell cycle distribution in tip and stalk cells were measured using flow cytometry. HUVEC were seeded (5000 cells/cm^2^) in 5 mL Endothelial Cell Growth Medium-2 in T-25 flasks (TPP). When the cells were approximately 70% confluent, they were treated in fresh complete medium containing DMSO (solvent control) or navtemadlin in the absence or presence of VEGF (20 ng/mL). After 22 h treatment, EdU (Click-iT™ EdU Alexa Fluor™ 647 Flow Cytometry Assay Kit, cat # C10634, Thermo Fisher Scientific) was added at a final concentration of 10 µM to the drug-containing medium. Following an additional incubation of 2 h, cells were harvested using DetachKit2 according to the manufacturer’s instructions and washed with 1% bovine serum albumin (BSA) in PBS. The samples were Fc blocked for 10 min at RT, dilution 1:400 in PBS. Extracellular staining with CD34, at a 1:50 dilution (clone 561, PECy7 conjugated, Biolegend) in 1% BSA in PBS was performed for 30 min on ice, in the dark [28, 29]. The cells were fixed for 30 minutes on ice, with Fix/Perm buffer (eBioscience TM Foxp3/ transcription factor staining buffer set, Invitrogen). The cells were washed with 1% BSA in PBS and stained extracellularly for p53 (1:50 dilution, clone DO-7, PE conjugated, Biolegend) and p21 (1:50 dilution, clone F-5, FITC conjugated, Santa Cruz Biotechnology) in Saponin perm buffer (Click-iT™ EdU Alexa Fluor™ 647 Flow Cytometry Assay Kit, cat # C10634, Thermo Fisher Scientific). The cells were washed with 1% BSA in PBS and resuspended in Saponin perm buffer before the click it reaction was initiated according to the manufacturer’s instructions (Click-iT™ EdU Alexa Fluor™ 647 Flow Cytometry Assay Kit, cat # C10634, Thermo Fisher Scientific). The cells were washed with Saponin perm buffer and resuspended in FxCycle^TM^ Violet stain (cat # F10347, Life Technologies) in Saponin perm buffer, at a 1:1000 dilution and incubated at room temperature for 30 min. Samples were acquired on FACSVerse^TM^ (BD) and analyzed with FlowJo v. 10 (Treestar).

#### RT-qPCR

We measured p53 transcriptional activity and the expression of genes related to tip and stalk cell identities following p53 activation using RT-qPCR. For p53 transcriptional activity, HUVEC were seeded (2 × 10^4^ cells in 1 mL/well) in a 12-well tissue culture dish (TPP), allowed to attach, transfected with DsiRNA for 24 h, and then treated with fresh EGM-2 medium containing either DMSO (<0.1%) or navtemadlin (1 µM) for 6 h to avoid potential confounding effects of cell death on p53 activity. For angiogenic gene expression, HUVEC were seeded (1 × 10^5^ cells in 2 mL/well) in a 6-well tissue culture dish (TPP), allowed to attach for 36 h, and treated with fresh medium containing either DMSO (<0.1%), navtemadlin (0.05 µM or 1 µM), or sunitinib (1 µM) in the absence or presence of VEGF (20 ng/mL) for 24 h. Following drug incubation, cells were lysed using TRIzol™ Reagent (300 µL/well, cat # MAN0001271, ThermoFisher). Lysates were flash frozen on dry ice and stored at −80 °C until further use. Total RNA was extracted using the Direct-zol™ RNA Miniprep Kit (cat #R2050, Zymo Research) and treated with DNAse I following kit instructions before conversion to cDNA (200–400 ng per reaction) using reverse transcription (iScript cDNA Synthesis Kit, cat #1708890, BIO-RAD) as per manufacturer’s instructions.

To detect changes in gene expression, qPCR was performed in a 96-well plate (cat #4346907, Applied Biosystems) using pre-designed PrimeTime qPCR primers (Integrated DNA Technologies, Supplementary Table 2) containing PowerTrack™ SYBR Green Master Mix (cat # A46012, ThermoFisher). Primer efficiency was confirmed to be within the range of 90–110% using the standard curve method. For experiments involving p53 transcriptional activity, 500 nM primer (*TP53, CDKN1A, MDM2, or B2M*) and 5 ng cDNA were combined per reaction well. PCR was performed using an annealing temperature of 58 °C for 40 cycles. For experiments involving tip and stalk cell genes, a final primer concentration of 300 nM and 4.5 ng cDNA was used. Four tip cell genes (*CXCR4*, *ANGPT2*, *CD34*, and *DLL4*) and three stalk cell genes (*HES1*, *FLT1* (which encodes VEGFR1), and *JAG1*) were selected based on prior studies. Touchdown PCR [49] was used to reduce the formation of primer dimers: for the first 10 cycles, the annealing temperature was 68 °C and reduced 1 °C per cycle until a final temperature of 58 °C was reached; for the next 30 cycles, the annealing temperature was set to 58 °C. The data were analyzed using StepOnePlus™ Software v2.3. Technical duplicates were averaged and normalized using *B2M* for the p53 transcriptional genes measured at 6 h, while they were normalized per plate using NORMA-Gene [50] for angiogenesis genes because all tested reference genes were affected by treatment at 24 h. The delta Ct values were calculated by subtracting the Ct value of control to that of treatment.

### In vivo experiments

#### Zebrafish embryo breeding

*Tg*(fli1:eGFP) zebrafish (*Danio rerio*) embryos were used as an in vivo model to evaluate the effect of p53 activation on sprouting angiogenesis of subintestinal vessels. Zebrafish were housed as previously described [51]. Health monitoring, performed by Charles River according to the FELASA-AALAS guidelines [52], detected the following: Mycobacterium chelonae in randomly sampled fish and in sludge samples, Mycobacterium fortuitum in sludge samples, and ZfPV-1 in sentinel fish, randomly sampled fish, and sludge. Zebrafish embryos were staged according to published guidelines [53]. All husbandry procedures were defined in standard operating protocols and are available with the health monitoring reports on request.

#### Angiogenesis assay in zebrafish embryos

A phenotypic toxicity assay was initially performed to obtain the maximum tolerated dose at which no toxic effect was observable in zebrafish embryos. The assay was performed in a 96-well plate under a stereomicroscope for the following compounds: sulanemadlin, control peptide, and sunitinib. The substances were tested at the following concentrations: 50, 25, 12.5, 6.25, 3.13, 1.56, 0.78, 0.39, and 0 µM. For each concentration of a compound, 5 embryos were assessed and scored for the following toxicity endpoints: number of clotted (dead) embryos, number of embryos lacking somite, number of embryos lacking a heartbeat, and number of embryos with heart oedema.

To measure the effect of p53 activation on angiogenesis in a venous bed, zebrafish embryos were dechorionized and incubated 20 h post-fertilization (hpf) in an exposure medium containing vehicle (DMSO 0.1%) or one of three compounds (sulanemadlin, control peptide, and sunitinib). The exposure medium contained 160 µg/mL tricaine and 30 µg/mL phenylthiourea in E3 medium. The embryos were then randomly distributed equally with the exposure medium in a 96-well plate (cat #89621, Ibidi) coated with agarose gel. The agarose gel was prepared using 1% agarose in 1xE3 medium. The plate was placed in the Image Xpress Nano (Molecular Devices) instrument for imaging at 48 h post-exposure. The images were taken using the GFP channel using the 4X objective (1.63 µm/pixel), stacked into a maximum intensity projection for each well, and exported as tif files. Images containing no embryos, blurry resolution of vessels, or embryos with anatomical deformities or spinal curvature were excluded prior to vessel segmentation. The subintestinal vessels were analyzed because they rely on VEGF to sprout from the cardinal vein around 30 hpf with both tip and stalk cells proliferating during formation. Masks of the subintestinal vessel area and total fish area were manually traced using ImageJ or Slicer.

### Statistical analysis

After data were evaluated for equal variance and for normality, statistical significance was evaluated using analysis of variance or non-parametric tests followed by multiple testing correction (α = 0.05). For recovery experiments, statistical significance was evaluated using a mixed-effects model with matching and a Geisser-Greenhouse correction, and then adjusted for multiple testing using Dunnett’s multiple comparison test. For quantification of immunofluorescence staining (p53, p21, Ki67, PUMA) and senescence staining, values were log-transformed and then evaluated for statistical significance using ANOVA, with matching across independent experiments, before adjustment for multiple testing using Dunnett’s multiple comparison test. For SytoxGreen assays, fluorescent intensity values were log-transformed and analyzed using a repeated measures ANOVA, followed by adjustment for multiple testing using Dunnett’s. For sprouting assays, measured parameters were log-transformed prior to analysis for statistical significance using the Kruskal-Wallis non-parametric test and corrected for multiple testing using Dunn’s multiple comparisons test. For permeability assays, fluorescent intensity values were log-transformed and analyzed using one-way ANOVA, followed by adjustment for multiple testing using Dunnett’s. For zebrafish assays, statistical significance of log-transformed values of vessel and fish areas was assessed using a Brown-Forsythe ANOVA followed by the Dunnett’s T3 multiple comparisons test, while that of sprout number was assessed using a Kruskal-Wallis non-parametric test following by Dunn’s multiple comparisons test. For flow cytometry experiments, statistical significance was evaluated using one-way or two-way ANOVA with matching followed by Dunnett’s multiple comparisons test. For RT-qPCR assays on knockdown cells, we evaluated statistical significance of the difference in Ct values using two-way ANOVA followed by Šidák’s correction. For angiogenesis genes, we evaluated significance using multiple unpaired t-tests followed by multiple testing correction using *FDR* < 0.01.

Individual data points represent averaged values of technical replicates from independent biological experiments; the number of biological replicates is reported in the figure legends. *P-*values were adjusted (*P*_*adj*_) as described above and are reported in the main figures and text. Sample sizes for cell assays were chosen based on prior experience and pilot experiments. Sample sizes for zebrafish assays were pre-determined to account for an effect size of ~30% and high biological variability, and for loss of embryos due to handling, drug effects, or image artifacts. Samples in cell assays were excluded if they failed quality control (e.g., poor RNA quality), while zebrafish samples were excluded if anatomical deformities or image artefacts were present. Randomization and blinding were not performed unless explicitly stated.

## Supplementary information

Supplementary Information

Full Length Western Blots

## Data Availability

The mass spectrometry proteomics data have been deposited to the ProteomeXchange Consortium via the PRIDE partner repository with the dataset identifier PXD060120. Other datasets generated during the current study are available in the Zenodo repository (DOI: 10.5281/zenodo.17324927). Any other relevant data are available upon reasonable request from the corresponding author.

## Code availability

Analysis scripts used in this study is available in Zenodo repository (DOI: 10.5281/zenodo.17324927). Image analysis on immunofluorescence stainings was performed using scripts written in Octave (v. 4.4.1) and ImageJ (1.51n). Statistical analyses for the migration assay and principal component analysis plots were generated using RStudio (v. 2022.02.3 build 492). Functional and protein networks were generated using Cytoscape (v. 3.9.1). Heatmaps of gene expression were generated using Python (v. 3.8.8) using the packages pandas, seaborn, and matplotlib.
