# Supplementary material for: Pharmacological activation of p53 induces dose-dependent changes in endothelial cell fate during angiogenic sprouting
Source: Cell Death Dis. 2025 Dec 8;16(1):883. doi: 10.1038/s41419-025-08292-7 (PMC12698774; doi:10.1038/s41419-025-08292-7)
Supplement: Supplementary file 1 — Supplementary Information [file 41419_2025_8292_MOESM1_ESM.pdf]

**SUPPLEMENTARY INFORMATION**

# Pharmacological activation of p53 induces dose-dependent changes in endothelial cell fate during angiogenic sprouting

Omayma Al-Radi[1], Katrine Ingelshed[1,2], Lisa Eichhorn[1], Heidi Josefsson[1], Martin Krkoska[1,3], Lars Bräutigam[4], Susanne Lindström[4], Ákos Végvári[5], Sania Kheder[1], Carmine P Cerrato[1], Suzon Fermé[1], Cecilia Bosdotter[1], Amin Allalou[6,7,8], Fredrik Levander[9], Borivoj Vojtesek[3], David P Lane[1], Pavitra Kannan[1]*

[1] Department of Microbiology, Tumor and Cell Biology, Karolinska Institutet, 171 77 Stockholm, Sweden

[2] Department of Immunology, Genetics and Pathology, Uppsala University, 75185 Uppsala, Sweden

[3] RECAMO, Masaryk Memorial Cancer Institute, 656 53 Brno, Czech Republic

[4] Comparative Medicine, Karolinska Institutet, 171 77 Stockholm, Sweden

[5] Department of Medical Biochemistry and Biophysics, Karolinska Institutet, 171 77 Stockholm, Sweden

[6] Department of Information Technology, Uppsala University, 751 05 Uppsala, Sweden

[7] DanioReadout, Immunology Genetics and Pathology, Uppsala University, Uppsala, Sweden

[8] SciLifeLab BioImage Informatics Facility, Uppsala University, Uppsala, Sweden

[9] Department of Immunotechnology, National Bioinformatics Infrastructure Sweden, Science for Life Laboratory, Lund University, Lund, Sweden

**Supplementary Table 1**. Information regarding the concentrations and source of antibodies used in western blotting (WB), immunofluorescence staining (IF), and flow cytometry (FC) assays.

| **Protein target** | **Assay** | **Catalog#** | **Company** | **RRID** | **Concentration used (µg/mL)** |
|---|---|---|---|---|---|
| p53 (clone DO-1) | WB | | in-house[1] | | 0.40 |
| MDM2 (clone IF2) | WB | 33-7100 | Invitrogen | AB_2533136 | 0.50 |
| p21 (clone 12D1) | WB | 2947 | Cell Signaling Technologies | AB_823586 | 0.24 |
| PUMA (clone D30C10) | WB | 12450 | Cell Signaling Technologies | AB_2797920 | 0.96 |
| TIGAR | WB | 22136-1-AP | Proteintech | AB_2879003 | 0.56 |
| TP53I3 | WB | 4828-1-AP | Proteintech | AB_2206893 | 0.45 |
| KPNA2 | WB | 10819-1-AP | Proteintech | AB_2265526 | 0.16 |
| PECAM | WB | 28083-1-AP | Proteintech | AB_2881055 | 0.09 |
| THBS1 | WB | 18304-1-AP | Proteintech | AB_2201959 | 0.40 |
| p53 (clone DO-1) | IF | | in-house[1] | | 12.00 |
| p21 (clone 12D1) | IF | 2947 | Cell Signaling Technologies | AB_823586 | 1.22 |
| Ki67 (clone SP6) | IF | ab16667 | Abcam | AB_302459 | 0.12 |
| PUMA (clone D30C10) | WB | 12450 | Cell Signaling Technologies | AB_2797920 | 4.80 |
| ZO-1 | IF | 21773-1-AP | Proteintech | AB_10733242 | 0.40 |
| hVE-cadherin | IF | AF938 | R&D systems | AB_355726 | 4.00 |
| PE/Cy7 anti-human CD34 (clone 561) | FC | 343615 | BioLegend | AB_2629725 | 1.00 |
| PE anti-human p53 (clone DO-7) | FC | 645805 | BioLegend | AB_2562267 | 0.24 |
| FITC anti-human p21 (clone F-5) | FC | sc-6246 | Santa Cruz Biotechnology | AB_628073 | 4.00 |

**Supplementary Table 2**. Sequences for human primers used in quantitative PCR to measure expression of genes involved in p53 activity and angiogenesis.

| Gene | Ref seq# | Forward primer | Reverse primer | Assay ID |
|---|---|---|---|---|
| *TP53* | NM_001276761 | TGACACGCTTCCCTGGATTG | GGCAAGGGGGACAGAACG | Hs.PT.58.39676686 |
| *CDKN1A* | NM_078467 | GCAGACCAGCATGACAGAT | GAGACTAAGGCAGAAGATGTAGAG | Hs.PT.58.40874346 |
| *MDM2* | NM_002392 | AGAAGGACAAGAACTCTCAGATG | GTGCATTTCCAATAGTCAGCTAA | Hs.PT.58.358457 |
| *HPRT1* | NM_000194 | CCTGGCGTCGTGATTAGTGAT | AGACGTTCAGTCCTGTCCATAA | Hs.PT.58v.45621572 |
| *B2M* | NM_004048 | GGACTGGTCTTTTCTATCTCTTGT | ACCTCCATGATGCTGCTTAC | Hs.PT.58v.18759587 |
| *CXCR4* | NM_003467 | AGCAGGTAGCAAAGTGACG | CCTCGGTGTAGTTATCTGAAGTG | Hs.PT.58.22298491 |
| *DLL4* | NM_019074 | CCAACTGCCCTTCAATTTCAC | GGATGGCGATCTTGCTGAT | Hs.PT.58.3416363 |
| *CD34* | NM_001773 | GACCTTTCAACCACTAGCACT | TGCCTGAACATTTGATTTCTGC | Hs.PT.56a.24708916 |
| *ANGPT2* | NM_001118888 | GTGCTGGAGAACATCATGGAA | GGTCCAAAATCTGTTTTTCCAAT | Hs.PT.560.27097148 |
| *FLT1* | NM_002019 | GCTCTCTATGAAAGTGAAGGCA | CATCCTCTTCAGTTACGTCCTT | Hs.PT.58.40906831 |
| *HES1* | NM_005524 | GAAATGACAGTGAAGCACCTC | TCACCTCGTTCATGCACTC | Hs.PT.58.4181121 |
| *JAG1* | NM_000214 | ACTATGCCTGTGACCAGAATG | CTTAGGACTGCAGCCTTGTC | Hs.PT.56a.4972610 |

## FIGURES

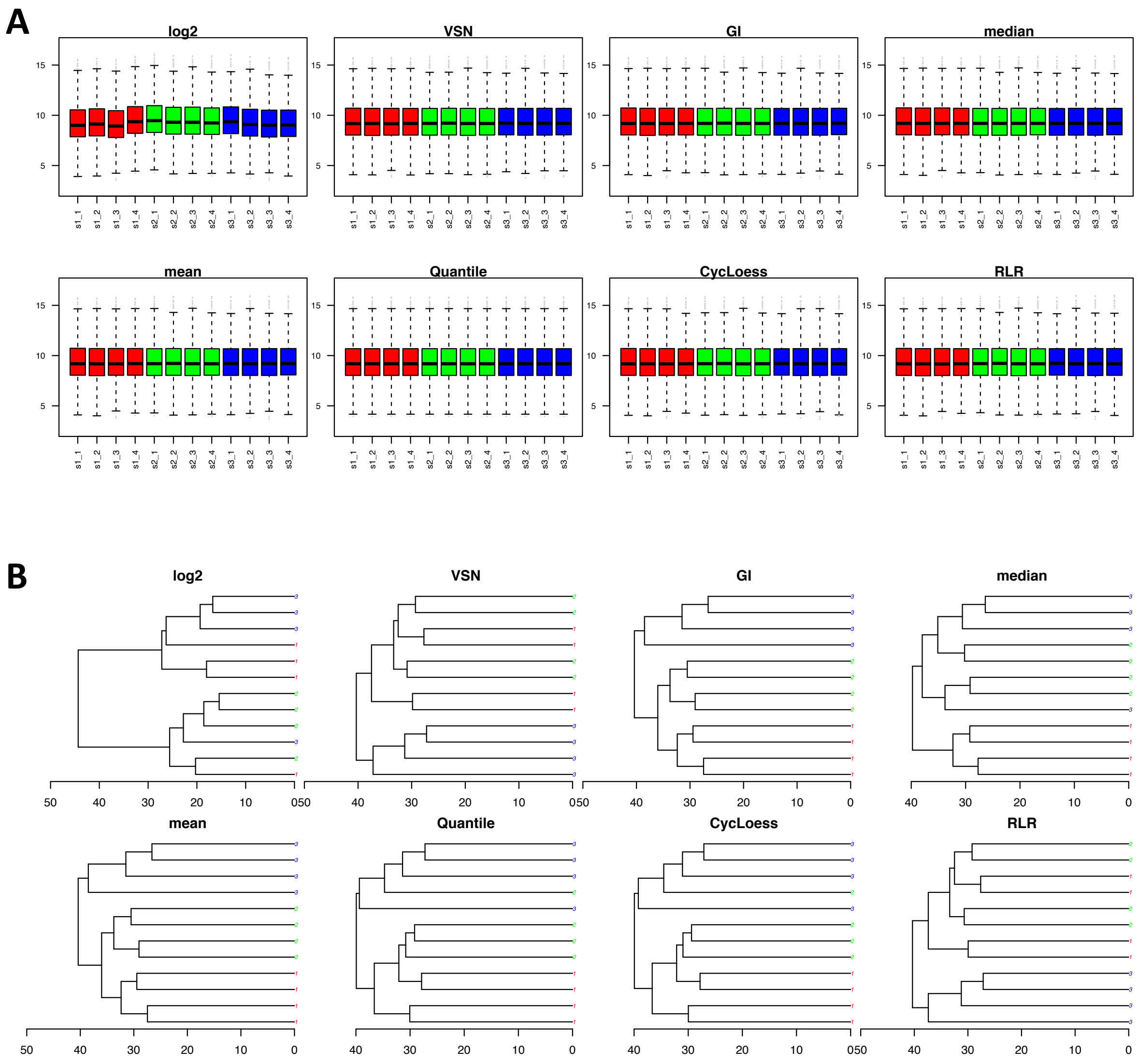


**SI Figure 1. Proteomics analysis in HUVEC lysates shows similar protein distribution across samples and best separation using mean normalized data. (A)** Boxplots of normalized protein abundances assessed using eight different normalization methods show consistent distributions across treatments. The normalization methods were: log2, variance stabilization normalization, generalized integration, median, mean, quantile, cyclic loess, and robust linear regression. Protein abundances were analyzed from lysates of human umbilical venous endothelial cells (HUVEC) treated with DMSO, 0.5 μM navtemadlin, and 1 μM navtemadlin. Colors represent different treatment groups. **(B)** Dendrogram separation of proteomics data normalized using 8 normalization methods show hierarchical clustering of post-normalized samples. Mean clustering showed best separation and was used for subsequent downstream analysis.

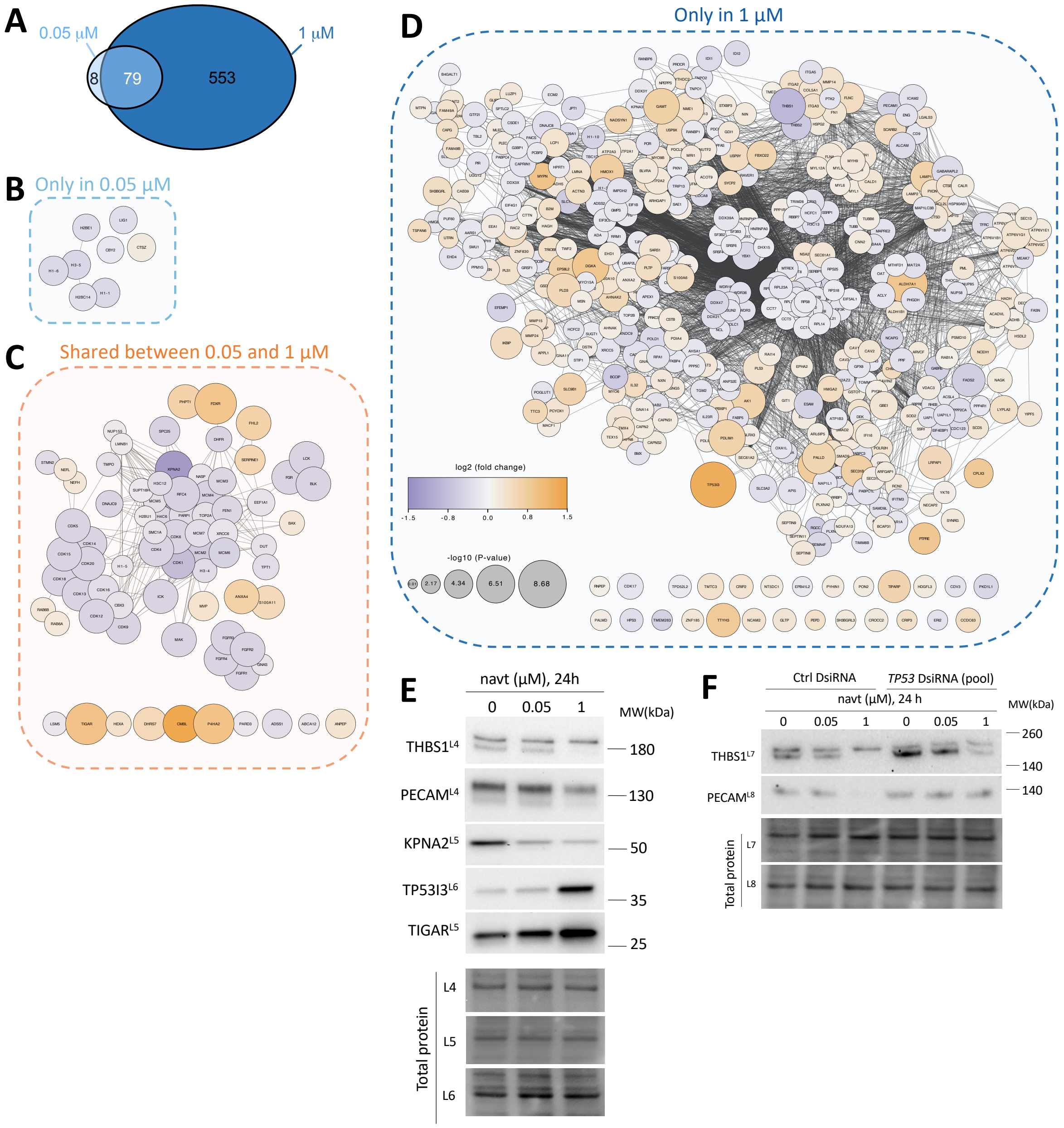


**SI Figure 2. Concentration dependent effects of p53 activation by navtemadlin on the proteome and protein-protein interactions in HUVEC. (A)** Venn diagram of differentially expressed proteins uniquely identified at low and high concentrations of navtemadlin, as well as those that were shared among the two concentrations. **(B-D)** Protein-protein interactions among differentially expressed proteins within the unique and shared protein sets, with color scale indicating log2 fold-change (purple = down; orange = up), and size of the node indicating adjusted -log10 P-value. **(E)** Expression of five select proteins (THBS1, PECAM, KPNA2, TP53I3, and TIGAR) that were differentially expressed in proteomics dataset, as assessed by Western blotting of HUVEC lysates treated using two different concentrations of navtemadlin for 24 h. **(F)** Rescued expression of THBS1 and PECAM following TP53-knockdown, as assessed by Western blotting of HUVEC lysates transfected using control or TP53-DsiRNA and then treated with navtemadlin for 24 h. Total protein levels show loading controls. Western blot images are cropped from full-length blots of one biological experiment (see ‘Full Length Western Blots’) and are representative of at least three biological experiments.

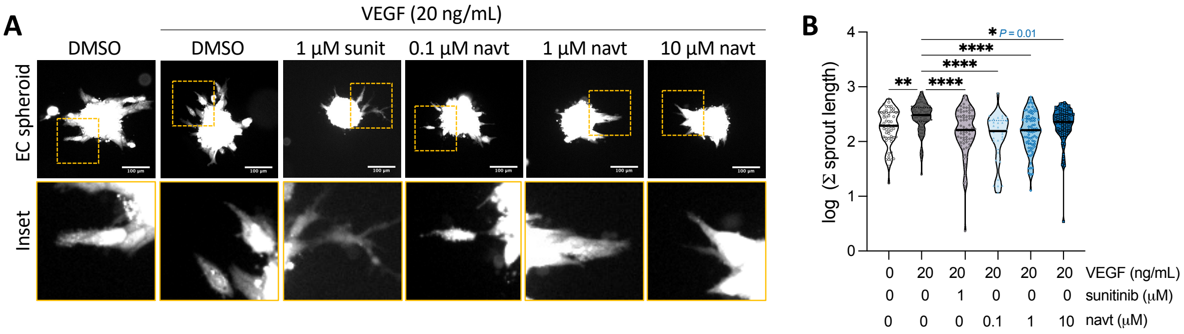


**SI Figure 3. Pharmacological activation of p53 reduces sprouting of spheroids comprising human dermal microvascular endothelial cells (HDMEC). (A)** Fluorescence microscopy images show reduced sprouting in HDMEC spheroids treated with increasing concentrations of sunitinib or navtemadlin in the absence and presence of vascular endothelial growth factor (VEGF). Insets show cell filipodia in sprouts. Scale bars = 100 µm. **(B)** All tested concentrations of navtemadlin reduce total sprout length of HDMEC spheroids. Each data point in violin plot indicates one spheroid (n = 60 spheroids for baseline; 92 spheroids for VEGF; 76 spheroids for sunitinib; 33 spheroids for 0.1 µM navt; 85 spheroids for 1 µM navt; 71 spheroids for 10 µM navt; pooled from two independent experiments). *$P_{adj} < 0.05$, ** $P_{adj} < 0.01$, ***$P_{adj} < 0.001$, **** $P_{adj} < 0.0001$ using Kruskal-Wallis with Dunn's correction. Note y-axis is shown on log scale.

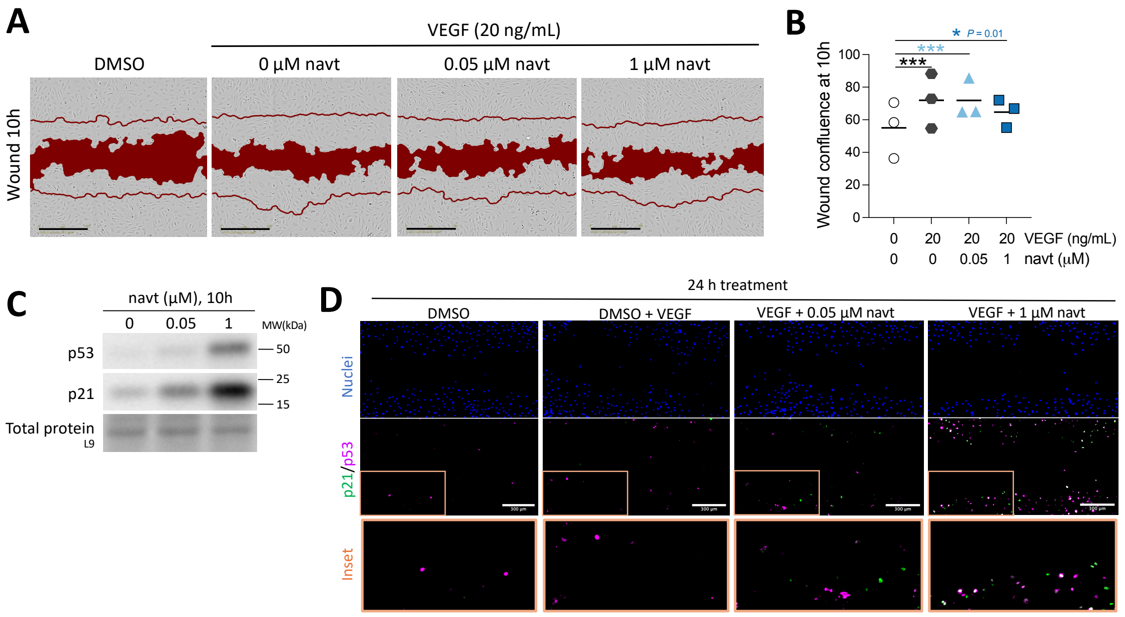


**SI Figure 4. Pharmacological activation of p53 by navtemadlin does not significantly reduce VEGF-induced migration of HUVEC. (A)** Images of migrating endothelial cells after 10 h treatment in a scratch wound assay. The thin red line demonstrates the size of the initial scratch, while the filled-in red area demonstrates the remaining plate surface that is not covered by endothelial cells. **(B)** Quantification of wound confluence at 10 h from the scratch wound assay**.** *$P_{adj}$ < 0.05, ***$P_{adj}$< 0.001, using a linear-mixed effects model with treatment as a fixed effect and experiment as a random effect to account for variability across different experimental runs. Horizontal black line shows mean value. **(C)** Expression of p53 and p21 increases in HUVEC lysates following 10 h navtemadlin treatment, as measured by western blotting. Images are cropped from full-length blots of one biological experiment (see ‘Full Length Western Blots’) and are representative of at least three biological experiments. **(D)** Increased expression of p53 and p21 in the scratch wound following 10 h navtemadlin treatment, as visualized by immunofluorescence staining. Nuclei are shown in blue. Scale bar = 300 µm.

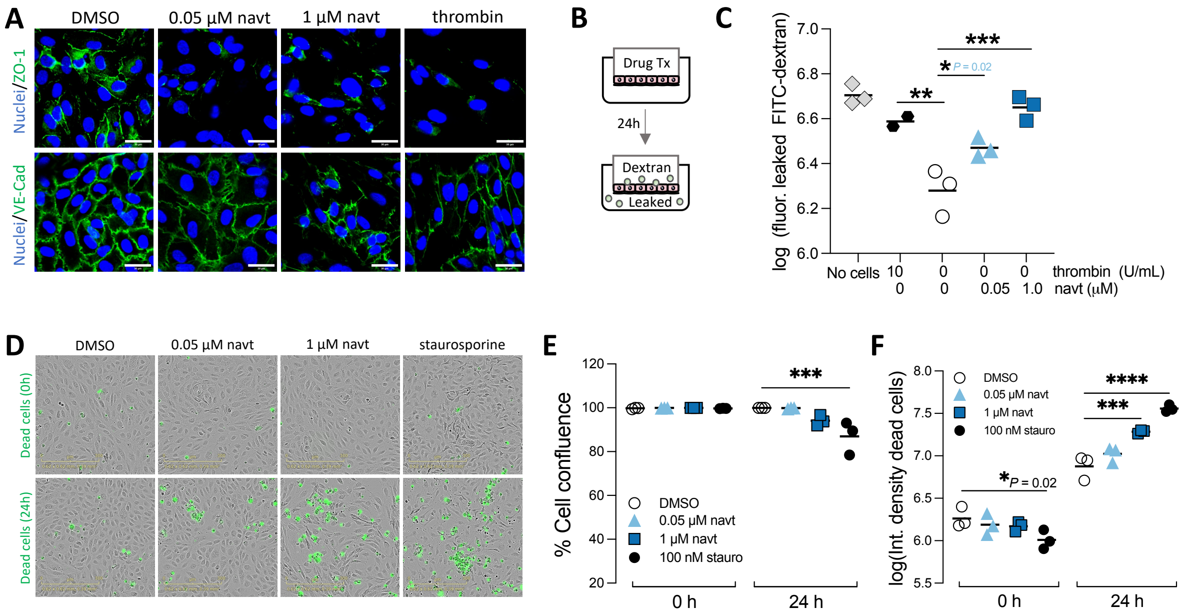


**SI Figure 5. Pharmacological activation of p53 by navtemadlin does not significantly reduce VEGF-induced migration of HUVEC.** **(A)** Reduced staining of tight junction markers zona-occludin 1 (ZO-1, green) and adherens junction marker VE-cadherin (VE-CAD, green) in HUVEC monolayers treated with navtemadlin (0.05 μM and 1 μM) and thrombin (positive control). Fluorescence images were acquired using confocal microscopy. Scale bar = 20 μm. **(B)** Schematic of *in vitro* permeability assay measuring FITC-dextran leakage across HUVEC monolayers after 24 h treatment. FITC-dextran was added to the upper compartment and fluorescence was measured after 30 min incubation from the bottom compartment. **(C)** Increased leakage of FITC-dextran across HUVEC monolayers following treatment with thrombin (positive control) or navtemadlin (0.05 μM and 1 μM). Symbols indicate averaged value from one experiment (n = 3 experiments for all except thrombin). Horizontal black line indicates mean value. *$P_{adj}$ < 0.05, ** $P_{adj}$ < 0.01, ***$P_{adj}$ < 0.001 using one-way ANOVA with Dunnett's correction for multiple testing. **(D)** Representative phase contrast images of cell death (as measured by SytoxGreen) in confluent HUVEC monolayers following 24 h treatment with DMSO, navtemadlin (0.05 μM and 1 μM) or staurosporine (100 nM). **(E-F)** Quantification of (E) cell confluence and (F) fluorescence intensity of dead cells within the treated confluent monolayers at 0 and 24 h of treatment. Fluorescence intensity is reported as the log of the integrated density values. ). Horizontal black line indicates mean value. *$P_{adj}$ < 0.05, ***$P_{adj}$ < 0.001 using two-way ANOVA with Dunnett's correction for multiple testing.

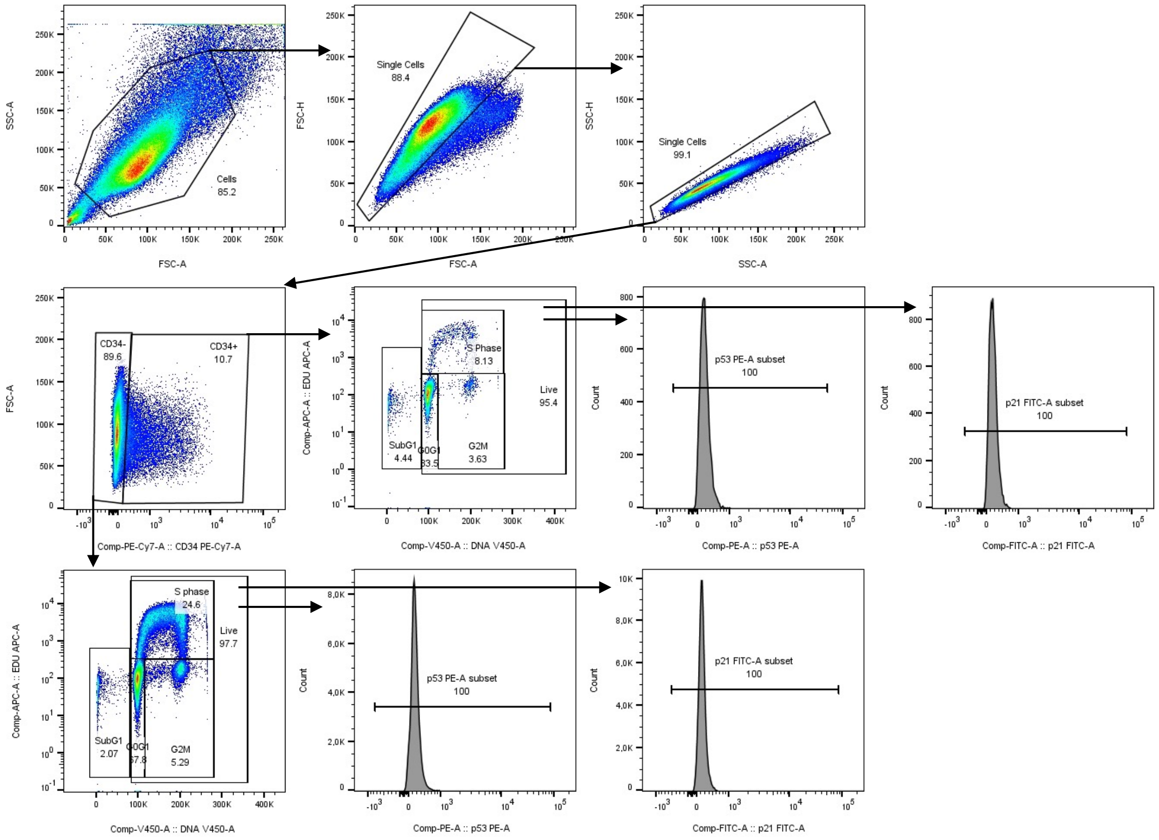


**SI Figure 6. Gating strategy for flow cytometry experiments for measuring tip cell frequency and cell cycle distributions in HUVEC.** After initial gating to exclude debris, and very large and granular cells, single cells were analyzed to identify tip cells (CD34+) vs non-tip cells (CD34-). Each population was subsequently analyzed for cell cycle phases and expression of p53 and p21.

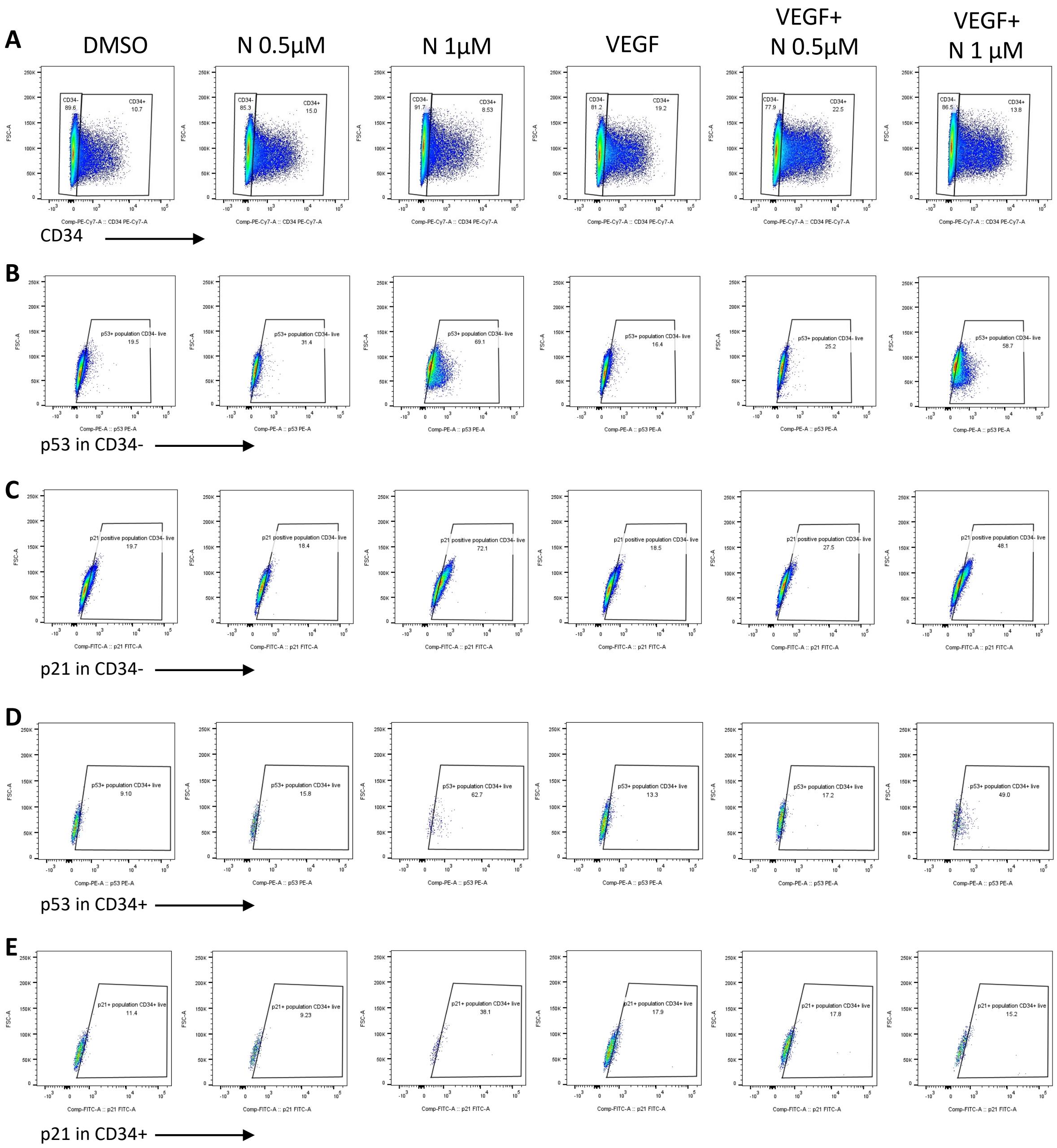


**SI Figure 7. Representative flow plots of HUVEC treated with navtemadlin in the absence and presence of VEGF. (A)** Representative plots of tip cell (CD34+) frequency in each treatment group. **(B-D)** Representative images of the frequency of (B) p53+ in CD34- cells, (C) p21+ in CD34- cells, (D) p53+ in CD34+ cells, and (E) p21+ in CD34+ cells. Full minus one (FMO) controls were used to correctly gate on positive populations. N = navtemadlin.

## REFERENCES

1 Vojtěšek B, Bártek J, Midgley CA, Lane DP. An immunochemical analysis of the human nuclear phosphoprotein p53. New monoclonal antibodies and epitope mapping using recombinant p53. *J Immunol Methods* 1992; **151**: 237–244.
