# Supplementary material for: Pharmacological activation of p53 induces dose-dependent changes in endothelial cell fate during angiogenic sprouting
Source: Cell Death Dis. 2025 Dec 8;16(1):883. doi: 10.1038/s41419-025-08292-7 (PMC12698774; doi:10.1038/s41419-025-08292-7)
Supplement: Supplementary file 2 — Full Length Western Blots [file 41419_2025_8292_MOESM2_ESM.pdf]

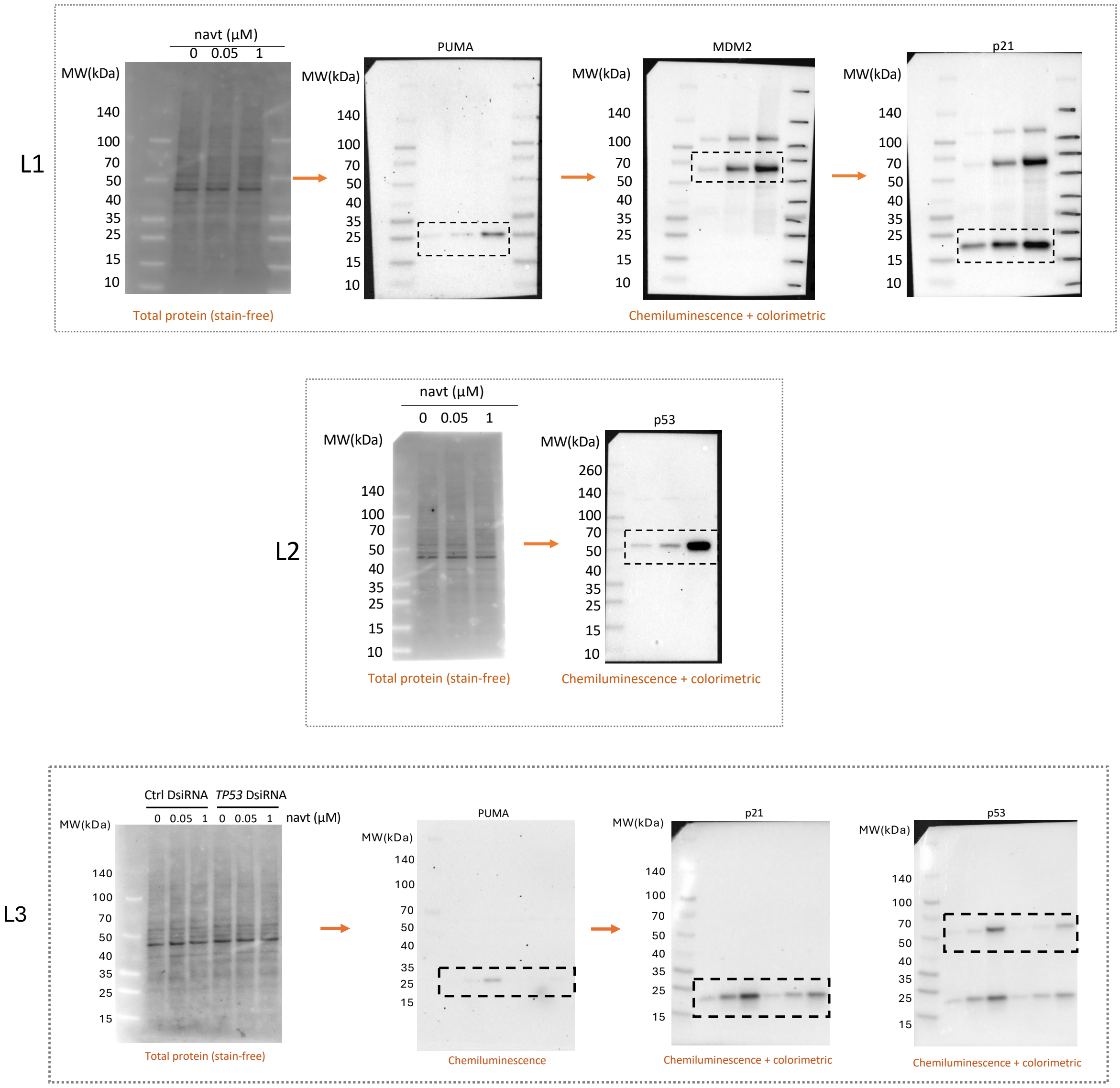

Whole blots from Figure 1E and Figure 2E. Blots showing total protein levels (stain-free) and target protein (merged chemiluminescence and colorimetric) detected using antibodies against: **(L1)** PUMA, MDM2, and p21, sequentially; **(L2)** p53; **(L3)** PUMA, p21, and p53, sequentially. Whole membranes were incubated with each antibody separately, imaged, and washed prior to addition of the subsequent antibody. Proteins were probed in the order indicated by the arrows. Concentrations of antibodies are shown in Supplementary Table 1.

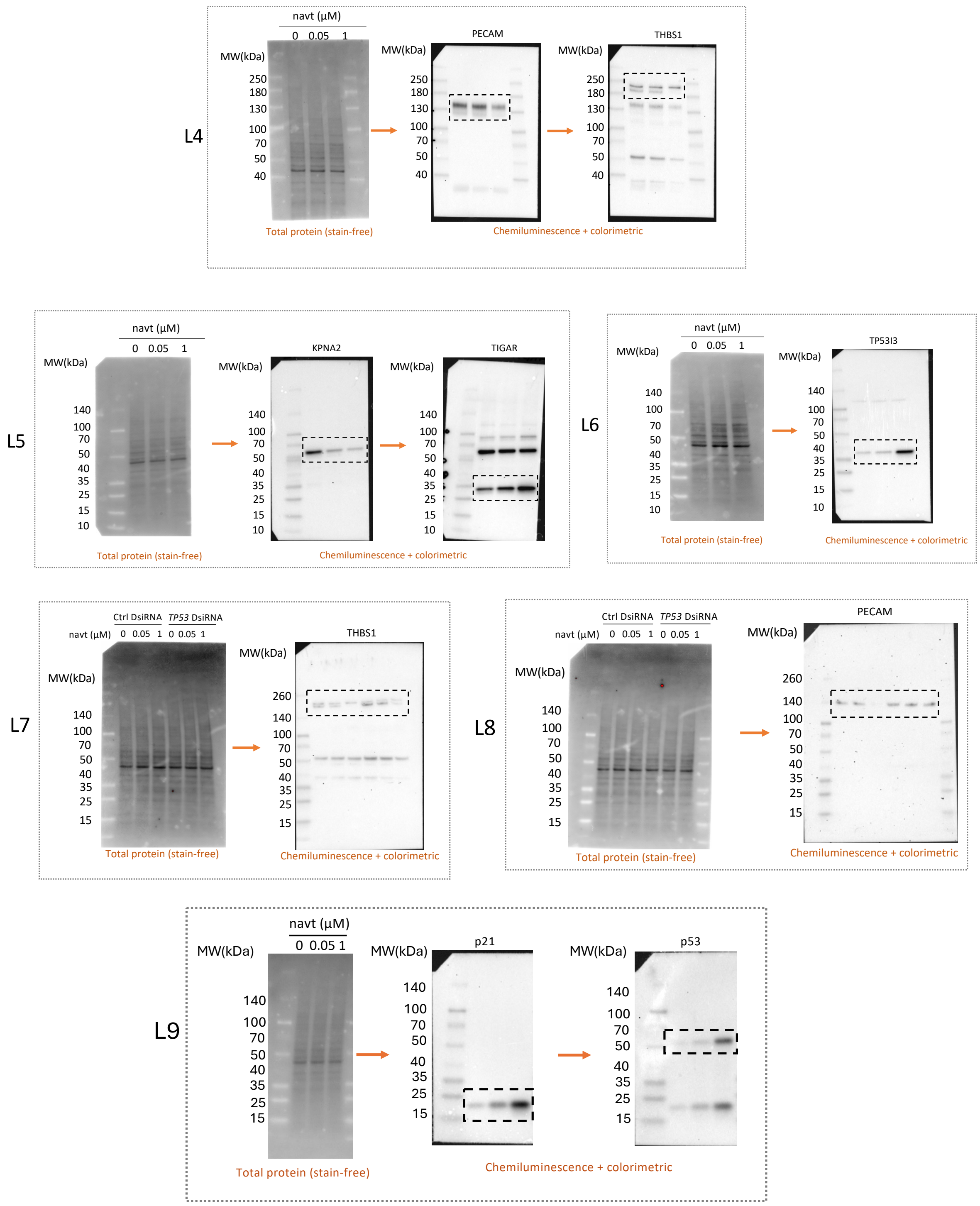

Whole blots from SI Figure 2E, SI Figure 2F, and SI Figure 4C. Blots showing total protein levels (stain-free) and target protein (merged chemiluminescence and colorimetric) detected using antibodies against: **(L4)** PECAM and THBS1; **(L5)** KPNA2 and TIGAR; **(L6)** TP53I3; **(L7)** THBS1; **(L8)** PECAM; and **(L9)** p21 and p53, sequentially. Whole membranes were incubated with each antibody separately, imaged, and washed prior to addition of the subsequent antibody. Proteins were probed in the order indicated by the arrows. Concentrations of antibodies are shown in Supplementary Table 1.
